# Pharmaceutical and Safety Profile Evaluation of Novel Selenocompounds with Noteworthy Anticancer Activity

**DOI:** 10.3390/pharmaceutics14020367

**Published:** 2022-02-06

**Authors:** Małgorzata Anna Marć, Enrique Domínguez-Álvarez, Gniewomir Latacz, Agata Doroz-Płonka, Carmen Sanmartín, Gabriella Spengler, Jadwiga Handzlik

**Affiliations:** 1 Department of Technology and Biotechnology of Drugs, Jagiellonian University Medical College, Medyczna 9, 30-688 Kraków, Poland; glatacz@cm-uj.krakow.pl (G.L.); a.doroz-plonka@uj.edu.pl (A.D.-P.); 2 Instituto de Química Orgánica General (IQOG-CSIC), Consejo Superior de Investigaciones Científicas, Juan de la Cierva 3, 28006 Madrid, Spain; 3 Department of Pharmaceutical Technology and Chemistry, School of Pharmacy and Nutrition, University of Navarra, 31008 Pamplona, Spain; sanmartin@unav.es; 4 Instituto de Investigaciones Sanitarias de Navarra (IdiSNA), 31008 Pamplona, Spain; 5 Albert Szent-Györgyi Health Center, Department of Medical Microbiology, Albert Szent-Györgyi Medical School, University of Szeged, Semmelweis utca 6, 6725 Szeged, Hungary; spengler.gabriella@med.u-szeged.hu

**Keywords:** selenoesters, ADMET, anticancer activity, pharmaceutical profile, PAMPA, metabolic stability, Ames test

## Abstract

Prior studies have reported the potent and selective cytotoxic, pro-apoptotic, and chemopreventive activities of a cyclic selenoanhydride and of a series of selenoesters. Some of these selenium derivatives demonstrated multidrug resistance (MDR)-reversing activity in different resistant cancer cell lines. Thus, the aim of this study was to evaluate the pharmaceutical and safety profiles of these selected selenocompounds using alternative methods in silico and in vitro. One of the main tasks of this work was to determine both the physicochemical properties and metabolic stability of these selenoesters. The obtained results proved that these tested selenocompounds could become potential candidates for novel and safe anticancer drugs with good ADMET parameters. The most favorable selenocompounds turned out to be the phthalic selenoanhydride (**EDA-A6**), two ketone-containing selenoesters with a 4-chlorophenyl moiety (**EDA-71** and **EDA-73**), and a symmetrical selenodiester with a pyridine ring and two selenium atoms (**EDA-119**).

## 1. Introduction

Despite of being poisonous at high doses, selenium is an essential trace element for living organisms [1]: it is involved in major metabolic pathways and crucial physiological functions, such as antioxidant defense and membrane stabilizing activity. Selenium plays a redox gatekeeper role in the detoxification and chemopreventive pathways in the human body [1,2]. Additionally, it can act as a free radical scavenger, antitumor, and antiaging agent [3]. Moreover, selenium is a micronutrient with potential applications in the treatment or prevention of specific diseases, including cardiovascular disorders, thyroid and neurodegenerative diseases, depression, acute pancreatitis (AP), viral infections (such as HIV), and cancer [4,5]. In all these pharmaceutical applications, the gap between toxic and therapeutic doses is narrow [6]. Furthermore, the most evident biological effect of selenium is the enhancement of the immune response, its antiviral activity, the regulation of thyroid hormones, and the prevention of coronary diseases [3,7]. Although selenium is beneficial at low concentrations, an excess of it can be toxic [8,9] and lead to severe disorders, such as selenosis in the most acute cases [1]. Regarding its deficiency, low serum selenium levels are linked to a higher risk of developing several cancer types, especially prostate, lung, and colorectal cancers [10]. For instance, a recent case-control study demonstrated a linear correlation between low selenium level and lung and laryngeal cancers, revealing that selenium levels below 60 μg/L were associated with a higher risk of developing lung or laryngeal cancer [11]. 

Several in vitro and in vivo experimental models have demonstrated the anticancer efficacy of selenocompounds (Se compounds), and several works have reviewed different selenium-containing derivatives with potential applications in cancer prevention or treatment [12,13,14,15,16]. Se compounds possess the ability to alter redox homeostasis and interfere with the cell signaling in cancer cells. One of these effects is the alteration of key regulatory elements of crucial cellular pathways, like the checkpoints of cell cycle, thus affecting the differentiation, proliferation, senescence, and cell death pathways [12]. Antioxidant and anti-inflammatory effects have also been observed. These functions are related to the antioxidant and pro-oxidant properties of Se compounds, which can oxidize sulfhydryl groups, alter the cellular thiolstat, cause DNA damage, and produce reactive oxygen species (ROS) [5,8,17]. 

Previous works of our group revealed that phthalic selenoanhydride and certain selenoesters showed a significant antiproliferative and/or cytotoxic activity, even at the nanomolar scale, against several cancer cells such as MCF-7 (breast cancer), HT-27 (colon cancer), A-549 (lung cancer), and PC-3 (prostate cancer), which are the most active ones; they were found to be significantly stronger than the reference compounds etoposide and cisplatin, which are widely used in cancer therapy [18]. Additionally, these tested Se compounds possess dual anticancer and chemopreventive activity [18]. The proposed mechanism of action underlying the activity of these Se compounds is the controlled hydrolysis of the selenoester or the selenoanhydride functionality [18,19]. This hydrolysis enables the release of selenium-containing anions or of reactive selenium species (RSeS) into the cellular environment. Therefore, these RSeS are ready to be involved in intracellular redox reactions, mainly with thiols, ROS, and free radicals. Accordingly, phthalic selenoanhydride can interact with glutathione and hydrogen sulfide, scavenge free radicals such as superoxide (O_2_^−^) and ˙cPTIO (2-(4-carboxyphenyl)-4,4,5,5-tetramethylimidazoline-1-oxyl-3-oxide), and cleave pDNA. Interestingly, the sulfur or oxygen isosteres of phthalic selenoanhydride do not show these activities on their own, highlighting the role exerted by the selenium atom in these experimental activities [20]. 

Due to these promising activities, selenoanhydride and the most active selenoesters have been studied more in-depth, and it has been found that they have antibacterial activity against *Staphylococcus aureus* and *Chlamydia trachomatis* [21], as well as antiviral activity against herpes virus [22]. They were found to be able to inhibit bacterial biofilm formation [22] and the bacterial efflux pumps involved in bacterial resistance to antibiotics [21]. Similarly, these Se compounds inhibited the ABCB1 efflux pump, commonly involved in the resistance developed by multidrug-resistant cancer cells towards chemotherapy [19]. These results suggested that this phthalic selenoanhydride and these selenoesters are very promising scaffolds in medicinal chemistry, the pharmaceutical profile and safety of which are discussed in this paper.

Pharmaceutical research is not limited to the search for novel active compounds. They also have to be safe and have an appropriate pharmaceutical profile. Hence, the estimation of pharmaceutical profile evaluation (ADME: Absorption, Distribution, Metabolism, Excretion) in tandem with safety studies (Tox, toxicity) is an essential part of the first steps of the search for potential drug candidates and of preclinical research. Summing up, the critical properties in drug discovery, which describes the ability of a compound to be an ideal drug candidate, are interesting biological activity; adequate absorption, distribution, metabolism, and elimination capabilities; and a low toxicity [23,24]. In fact, the physicochemical properties of any novel potential drug candidate should be characterized. These properties include solubility, lipophilicity, and chemical stability in acidic and alkaline environments or buffers used in biological assays [25]. 

**Figure 1:**
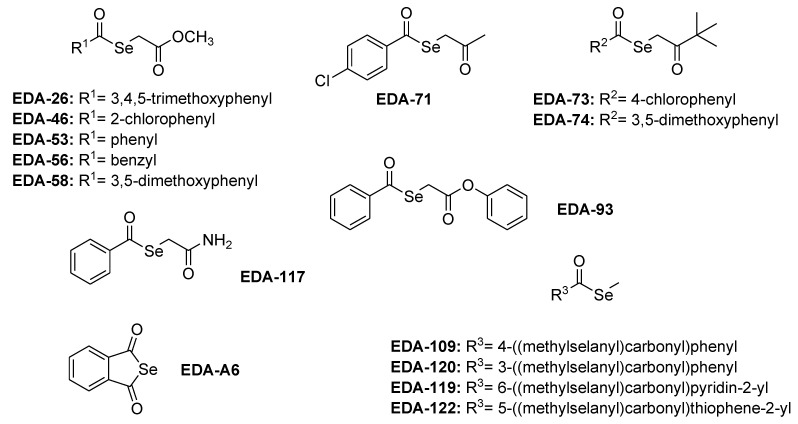
Tested selenocompounds with significant anticancer activity.

Here, we explore the key ADMETox parameters for the 15 most promising selenoesters and selenoanhydrides (Figure 1), according to the biological activities assessed in previous works [18,19,20,21,22]. 

**Figure 2:**
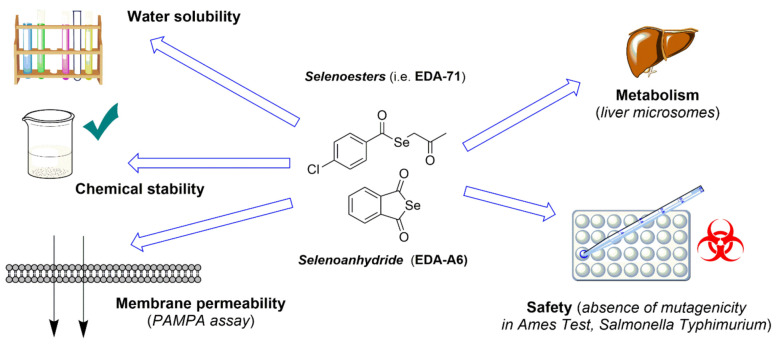
ADME and safety experiments carried out in this work for specific selenocompounds.

The selected in vitro assays cover fundamental physicochemical and biochemical properties of a drug candidate, such as solubility, cell membrane permeability (transporter effects), chemical and metabolic stability (specifically in the evaluation in human liver microsomes—HLMs), safety, and toxicology. These tests are essential screening elements during the drug discovery process focused on the search for new anticancer agents [26]. These assays are schematically presented in Figure 2.

The safety and pharmaceutical profiles of the tested Se compounds were estimated using alternative methods in vitro. One of the main tasks of this work was to determine selected physicochemical properties that affect the “druglikeness” of the tested Se compounds. These selected properties are the aqueous solubility, chemical stability in acidic and in alkaline solutions, and cell membrane permeability (determined with a PAMPA test). The metabolic stability of selected compounds was also determined using in vitro and in silico methods. The safety of the compounds was evaluated with the Ames test, following a protocol adapted to microplates that used the *Salmonella enterica* serovar Typhimurium (onwards *Salmonella* Typhimurium) TA 100 strain. 

This TA100 strain was selected due to its specificity and sensitivity for a wide range of mutagens. The TA100 strain is characterized by the base pair substitution *hisG46* mutation, which targets GGG [27,28]. The *Salmonella* Typhimurium TA100 strain has a base-pair substitution that can be reverted by mutations at GC pairs. In addition, it contains mutations at the *uvrB-bio* and *rfa* genes. Thanks to these mutations, the excision repair mechanisms are deactivated and the bacteria become more permeable to chemicals, respectively. Lastly, TA100 also includes the pKM101 plasmid, which is used to enhance both chemical and UV-induced mutagenesis and to increases the error-prone recombinational DNA repair pathway [29,30,31]. This strain of *Salmonella* Typhimurium is not able to produce histidine. Through mutagenic events, this can be reversed by means of reverse mutations, thus allowing for reverted bacteria to grow in histidine-deficient media and enabling the synthesis of histidine from the glucose present in the growth medium [32].

## 2. Materials and Methods 

### 2.1. Tested Organic Selenocompounds

The synthesis of the 15 selenocompounds evaluated in this work was previously reported [18]. All Se compounds were resynthesized and obtained with a high enough purity for their biological screening, i.e., their purity (assessed using microanalysis) was higher than 95%. Out of these 15 seleno-organic compounds (Figure 1), one was cyclic selenoanhydride benzo[*c*]selenophene-1,3-dione (**EDA-A6**, phthalic selenoanhydride), and the 14 remaining derivatives were selenoesters: methyl 2-((3,4,5-trimethoxybenzoyl)selanyl)acetate (**EDA-26**), methyl 2-((2-chlorobenzoyl)selanyl)acetate (**EDA-46**), methyl 2-(benzoylselanyl)acetate (**EDA-53**), methyl 2-((2-phenylacetyl)selanyl)acetate (**EDA-56**), methyl 2-((3,5-dimethoxybenzoyl)selanyl)acetate (**EDA-58**), *Se*-(2-oxopropyl) 4-chlorobenzoselenoate (**EDA-71**), *Se*-(3,3-dimethyl-2-oxobutyl) 4-chlorobenzoselenoate (**EDA-73**), *Se*-(3,3-dimethyl-2-oxobutyl) 3,5-dimethoxybenzoselenoate (**EDA-74**), phenyl 2-(benzoylselanyl)acetate (**EDA-93**), *Se*,*Se*-dimethyl benzene-1,4-bis(carboselenoate) (**EDA-109**), *Se*-carbamoylmethyl benzoselenoate (**EDA-117**), *Se*,*Se*-dimethyl pyridine-2,6-bis(carboselenoate) (**EDA-119**), *Se*,*Se*-dimethyl benzene-1,3-bis(carboselenoate) (**EDA-120**), and *Se*,*Se*-dimethyl thiophene-2,5-bis(carboselenoate) (**EDA-122**) [18]. 

### 2.2. Reagents and Equipment

The complete list of the reagents and the equipment used in this work is shown in the Supplementary Materials. 

### 2.3. Determination of the Aqueous Solubility

The solubility of all tested Se compounds was evaluated by means of in silico and in vitro methods. The solubility determination using in silico methods was performed with the following three computer programs: Chemicalize (https://chemicalize.com) [33], SwissADME (http://www.swissadme.ch) [34], and OSIRIS (http://www.organic-chemistry.org/prog/peo) [35]. 

Experimental aqueous solubility was determined following a previously described procedure [36,37]. Briefly, calibration curves were obtained by means of UV spectrometry for each compound, and their respective linear equations were obtained. Afterwards, saturated solutions of each compound were obtained, and they were diluted until the concentration fell within the calibration range, thus enabling the correct concentration interpolation. The multiplication by the dilution factor allowed us to determine the compounds’ solubility as the average of three independent experiments. A detailed description of this process can be found in Supplementary Materials. 

### 2.4. Chemical Stability in the Acidic and Alkaline Environments

The chemical stability of the new organic selenocompounds was evaluated in two different environments (acidic and alkaline). Firstly, tested selenocompounds were dissolved in pure anhydrous methanol to obtain a stock solution with a concentration of 2 mg/mL. Then, the solution was divided into two equal parts. A 10% solution of HCl was added in 1:1 proportion to the first part, and a 10% solution of NaOH was added to the second part in the same 1:1 proportion. Then, the stability of the tested Se compounds, either in the acidic or in the basic conditions, was TLC-monitored at specific time periods: 10 min and 1, 2, 3, 24, and 48 h. The following eluents were used: (i) methylene chloride for **EDA-26**, **EDA-73**, **EDA-74**, **EDA-93**, **EDA-109**, **EDA-119**, **EDA-120**, and **EDA-122**; a mixture of *n*-hexane and ethyl acetate in a 4:1 ratio for **EDA-58**; (ii) chloroform for **EDA-71** and **EDA-A6**; and (iii) acetone for **EDA-117**. For each tested compound, the retention factor (R_f_) was calculated at established times (10 min and 1, 2, 3, 24, and 48 h, in both acidic and alkaline stability experiments. The decomposition of a certain compound was observed when, in its TLC monitoring, an additional point appeared or the R_f_ value was significantly different from the one determined for the pure compound before the experiment. 

### 2.5. PAMPA Test: Membrane Permeability Evaluation

**Scheme 1:**
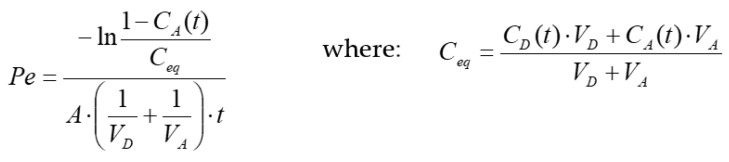
Calculation of the in vitro permeability (cm/s). *A* (filter area) cm^2^ = 0.3; *V_D_* (donor well volume) mL = 0.3; *V_A_* (acceptor well volume) mL = 0.2; *t* (incubation time) seconds = 18,000; *C_A_(t)* (compound concentration in acceptor well at time t); *C_D_(t)* (compound concentration in donor well at time *t*).

The passive transport and membrane absorption of 5 selected selenocompounds (**EDA-58**, **EDA-71**, **EDA-119**, **EDA-122**, and **EDA-A6**) were evaluated with the in vitro parallel artificial membrane permeability assay (PAMPA). This PAMPA assay could provide better predictability and membrane permeability correlations compared to the data obtained for human absorption in the Caco-2 cell line [38,39]. The Precoated PAMPA Plate System Gentest™ (Corning, Bradford, MA, USA) was used to perform the permeability assay for the 5 selected Se compounds. The protocol of the permeability assay was similar to the previously described ones [38,40,41] and is detailed in the Supplementary Materials. Briefly, the concentrations of the Se compounds and the references (norfloxacin and caffeine) were calculated in both the donor and acceptor compartments using capillary electrophoresis, which enabled the calculation of the permeability coefficients (Pe, (cm/s)) of the tested compounds with the formula provided by the PAMPA Plate System manufacturer [38,41]; see Scheme 1. The obtained results were compared with data regarding a highly permeable reference drug and a lowly permeable reference drug (caffeine and norfloxacin, respectively).

### 2.6. Metabolic Stability Evaluation

#### 2.6.1. In Silico Metabolic Stability Simulation (MetaSite)

The commercially available MetaSite 5.1.1 software has been used to evaluate the in silico simulation of liver metabolic pathways of selected organic selenocompounds. The use of this software is an easy computational method that can be used to perform the in silico prediction of the most probable sites of tested compounds’ structure that undergo metabolic reactions through the metabolic pathways related to the CYP450 reactions (e.g., CYP3A4) [42,43]. The MetaSite 5.1.1 software was provided by Molecular Discovery Ltd (c/o Sobell Rhodes LLP The Kinetic Centre, Hertfordshire, UK)., www.moldiscovery.com. This software enables the in silico prediction of the metabolism sites in the phase I reactions and therefore could be used in the preliminary assessment of the metabolic pathways. MetaSite can estimate metabolic routes in liver and cytochrome computational models. This program identifies the sites of the structure of the examined compounds that are most likely to suffer metabolic reactions (according to these computational models) while also predicting the structures of metabolites according to both a thermodynamic simulation (the enzyme–substrate recognition) and a kinetic simulation (the description of the enzyme-catalyzed chemical transformations) [44].

#### 2.6.2. In Vitro Microsomal Biotransformation Tests

The assessment of the metabolites generated in the in vitro microsomal biotransformation of the selenoester **EDA-71** in liver microsomes was performed following a previously described protocol with minor modifications [44,45]. Only this compound was evaluated because it combined an optimal solubility to be analyzed in this procedure with very significant activity. The protocol is provided in full detail in the Supplementary Materials. Briefly, the compound was exposed to microsomes for 2 h, and the mixture was later analyzed with LC–MS/MS to detect the generated metabolites. 

### 2.7. Safety Profile Evaluation—Ames Microplate Mutagenicity Assay

Safety profiles were evaluated using an *in vitro* microbiological method adapted to the microplate Ames test (384-well microtitration plate), which applies *Salmonella enterica* serovar Typhimurium bacterial strains (the gold standard in mutagenicity research). The experiment was performed according to a previously described protocol [45], which is provided in the Supplementary Materials. Briefly, the TA 100 strain (deprived of the ability to synthesize the histidine) was incubated with each tested compound in an exposure medium containing a limited amount of histidine. The reversion events were counted both manually and by means of a microplate reader [45]. Each fold increase bigger than two times the baseline level (≥2.0-fold) was considered to indicate a mutagen alert. The experiments and data analyses were prepared according to the procedure that was previously described by Flückiger-Isler et al. [46,47,48,49]. The assay was performed in triplicate. The positive control selected for the mutagenicity assays was NQNO, which causes genome point mutations; specifically, it promotes G:C → A:T transitions in the *Salmonella* Typhimurium TA-100 strain and other bacteria such as *Vibrio harveyi* and *Escherichia coli* [32,50].

## 3. Results and Discussion

### 3.1. Determination of Aqueous Solubility

In this work, special attention was paid to the solubility of the compounds because it is a crucial parameter in the determination of drug bioavailability and pharmacological activity. 

In the first stage of the research, the water solubility parameter (logS_m_) of the selenoanhydride and 10 selected selenoesters was evaluated in silico with the use of the Chemicalize [33], SwissADME [34], and OSIRIS [35] programs. All of the tested organic selenocompounds exhibited a very low water solubility according to the in silico methods (Table 1). It was observed that the solubility results obtained by the means of the SwissADME program were similar to those found with the OSIRIS computational method. The Chemicalize solubility values slightly differed from those obtained in the two abovementioned techniques, as they were significantly higher (Table 1). 

The aqueous solubility of the tested organic Se compounds was experimentally determined following a previously described procedure [36,37]. The chosen assay used UV spectrophotometry to estimate the concentration of the saturated solution with known dilutions of the tested compound at room temperature (20° C) after 24 h. The concentrations of the solutions were used to obtain the interpolation of the measured UV absorbance of the saturated solution dilution from the calibration curve drawn using solutions previously prepared in methanol/water mixtures.

The obtained experimental solubility values for the organic selenocompounds ranged from 0.001 to 1.028 g/L and were considered very low. The compound with an amide group in the alkyl moiety bound to the selenium atom of the selenoester (**EDA-117**) was the most soluble among the tested selenocompounds, displaying a 1.028 g/L solubility value. In contrast, the ketone-containing selenoester **EDA-73** was the least soluble, with a solubility of barely 0.001 g/L, more than 1000-fold less soluble than the most soluble Se compound (**EDA-117**). Summing up, according to the Biopharmaceutical Classification System [51,52], all the tested selenocompounds in this work could be classified as low-solubility derivatives. The obtained experimental logS_m_ values significantly differed from the in silico ones in most cases (Table 1). Significant similarity was only found in the case of **EDA-73** (−5.50_exp_. vs. −5.59_SwissADME_ and −5.82_Chemicalize_) and **EDA-122** (−4.02_exp_. vs. −3.76_Chemicalize_).

The experimental solubilities were higher than those predicted in silico for these compounds, with the exception of the symmetrical selenodiesters **EDA-109**, **EDA-119**, **EDA-120**, and **EDA-122** in Chemicalize. It was observed that this program predicted lower solubility values than the experiment for compounds with one selenium atom and a higher values for symmetrical compounds with two selenium atoms. Among the three in silico methods, the one that provided the closest results to the experimental ones was Chemicalize. After calculating the solubility values and comparing them to the experimental solubility values (data calculated from Table 1), the values were found to be on average 88.5% lower for mono-selenated derivatives and 210% higher for di-selenated compounds. The average prediction for all compounds in SwissADME and Osiris was 87.6% and 98.4% lower, respectively, than the experimental values. In the case of SwissADME, this average was not accurate, as it presented a very good approximation for **EDA-73** (the in silico value was only 18.7% lower than the experimental value) but the error in the remaining ones was very high (99.20% lower than the experimental value—almost two magnitude orders). In all cases, the error was too high, and these programs may not be adequate for evaluating the physicochemical properties of selenocompounds in silico. A possible reason for this is that the selenium atom is not well-parametrized in these programs, perhaps due to a lack of experimental data for selenocompounds. The different range of solubility for compounds with one or two selenium atoms in Chemicalize supports this hypothesis. This fact should be taken into account in future works, as it raises doubts regarding the accuracy of the predictions given by these programs for selenocompounds. A careful search was conducted in PubMed, and we did not find any additional studies that compared the in silico solubility determined by this program with experimental in vitro solubility, as performed in this study.

The low solubility was not a surprise: according to the literature, the majority of the synthetic organic selenocompounds are poorly soluble in water [53]. For example, methylseleno-aspirin showed a water solubility of only 2.20 × 10^−4^ M [54] (or 0.0568 g/L when taking its molecular weight into account). This fact suggests a lower effectiveness and higher risk of developing toxic side effects, as orally administered but poorly soluble compounds require higher doses to show desired activity [55]. To improve the solubility of water-insoluble anticancer drug candidates, the use of solubilizers, such as surfactants, organic solvents, cyclodextrins, lipids, and pH modifiers, is a common approach. Even though lowly water-soluble drugs can become moderately soluble if a suitable amount of additives is used, the usage of the additives need to be kept below their toxicity rate. Moreover, the usage of solubilizers can cause drug instability and is limited due to undesirable effects.

Sometimes, a chemical modification introduced to increase water solubility produces a decrease in drug potency. Similarly, modifications that improve in vitro cytotoxic actions cause significant solubility decreases. One of ways to avoid these undesired effects is preparing drug formulations in the form of drug delivery systems (DDSs), which include nanosuspensions, which were developed to increase the bioavailability of poorly water-soluble drugs. Additionally, drug delivery systems such as liposomes, polymer micelles, and dendrimers are broadly investigated and commonly used [56,57].

### 3.2. Chemical Stability in the Acidic and Alkaline Environments

In the next step of the experiments, the chemical stability of 11 selenoesters (**EDA-26**, **EDA-71**, **EDA-73**, **EDA-74**, **EDA-58**, **EDA-93**, **EDA-109**, **EDA-117**, **EDA-119**, **EDA-120** and **EDA-122**) and the selenoanhydride **EDA-A6** in acidic and alkaline environments was evaluated by the use of a previously described method [36,37]. Unfortunately, it was not possible to estimate the chemical stability of the compounds **EDA-46**, **EDA-53**, **EDA-56**, and **EDA-58** due to their physicochemical properties: they were liquid at room temperature in the form of an oil, which is not miscible with water. 

The majority of the tested Se compounds were unstable in the alkaline conditions. However, in the acidic environment, the results greatly varied regarding the function of the different groups present in the chemical structure. Some compounds, such as the selenoanhydride or **EDA-71,** were unstable at low pH environments, whereas others showed partial or total stability in these acidic conditions. The thiophene-containing diselenodiester **EDA-122** and its close analogue **EDA-119** (in which the thiophene moiety was replaced with a pyridine ring), **EDA-117**, and **EDA-58** were moderately stable (for 3 h) in the acidic media and totally unstable in the alkaline environment.

The ketone-containing selenoesters **EDA-73** and **EDA-74** were stable till the end of the experiment (48 h) in the acidic conditions and unstable in the alkaline conditions. Interestingly, the **EDA-71** analogue, which also belongs to the group of ketone-containing selenoesters, was totally unstable in the acidic environment—during the experiment, we observed a red precipitate product that was probably selenium oxide. Like the previously tested derivatives, **EDA-71** was unstable in the alkaline conditions. 

Finally, the selenoanhydride **EDA-A6** and selenoester **EDA-93** were proven to be unstable in both the acidic and alkaline environments. Moreover, compounds **EDA-26**, **EDA-109,** and **EDA-120** were stable till the end of the analysis (48 h) in the acidic conditions and totally unstable in the alkaline environment. 

Summing up, the selenoesters were generally moderately stable in the acidic conditions. However, after the addition of the 10% NaOH solution, very fast degradation was observed in the most cases. The most probable mechanism of this process is the hydrolysis of the selenoesters in the alkaline conditions. According to our knowledge, there have been no other studies on the chemical stability of small molecules-containing selenium and designed as novel bioactive compounds in acidic or alkaline environments. 

### 3.3. PAMPA Test: Membrane Permeability Evaluation

Each drug must overcome some obstacles after its administration, such as issues with absorption, and interactions with other compounds present in the diet or administered as therapeutic agents. Their distribution between various tissues is also important because it decides whether a compound can reach its target. Therefore, the determination of the tested compounds permeability through cell membranes is a crucial step in preclinical research because a low permeability can limit their activity, leading to lower drug effectiveness. 

The in vitro PAMPA screening test is one of the most important methods in the early stages of drug development. This method enables the estimation of the in vitro permeability of new drug candidates, as it simulates the structure and biological environment of the cell membrane. Besides, it enables the simple and straightforward estimation of a compound’s passive transport through biological membranes, characterized as a permeability coefficient P_e_ (in the unit of 10^−6^ (cm/s)) [38]. Depending on the nature of the artificial membrane, different biological barriers (intestinal or blood–brain) can be targeted [58].

The aim of the PAMPA test is to investigate in vitro membrane permeability via an estimation of the concentration of the tested compounds in donor and acceptor compartments separated by a filter membrane, which has a composition similar to the membrane of living cells [59]. Tested compounds are placed in the donor compartment, and then, after scheduled incubation at room temperature, their concentration in both compartments is determined using analytical methods. Here, to estimate the concentration of the compounds, capillary electrophoresis (CE) was used [38,41]. The PAMPA method can be performed at the small scale in the laboratory conditions. One of its limitations is the underestimation of the absorptions of compounds that are substrates of drug transporters. However, PAMPA may serve as a useful initial permeability evaluation in initial stages of the drug discovery process due to its high-throughput adaptability [60]. 

Five selenocompounds (**EDA-58**, **EDA-71**, **EDA-119**, **EDA-122**, and **EDA-A6**) were evaluated with the PAMPA in vitro assay using the CE technique. The obtained results were compared with data regarding two reference drugs: the high-permeability caffeine and the low-permeability norfloxacin [61,62]. Compounds were considered to be highly permeable when the experimentally estimated in vitro permeability value (P) was higher than 1.5 × 10^−6^ (cm/s). If the permeability value obtained with the PAMPA test was equal or lower than 1.5 × 10^−6^ (cm/s), the compound was considered lowly permeable through the cell membranes of enterocytes [38,39,40]. Results are shown in Table 2.

According to the results, all tested selenocompounds could be considered highly permeable. Selenoesters **EDA-122 and EDA-71** and the cyclic selenoanhydride **EDA-A6**, especially, showed higher permeability values than caffeine, whereas **EDA-119** and **EDA-58** showed lower permeability values than caffeine, though they were still in the same magnitude order. 

These results are noteworthy as they support the use of these compounds as pro-drugs that can serve to transport selenium inside cells, where the compounds can release, through hydrolysis, selenium anions or reactive selenium species that can then interact with cellular targets, as previously hypothesized [18,19] and experimentally observed for the case of the selenoanhydride **EDA-A6** [20]. The authors of a previous study [63] evaluated the permeability of different known selenocompounds and found that the most permeable ones were methylselenocysteine (MeSeCys) and selenomethionine (SeMet), with permeabilities of 17.2% and 12.4%, respectively. The other studied compounds were selenate (6.6%), anionic selenocyanate (3.8%), selenite (3.4%), selenocystine (3.1%), cationic trimethylselenonium (3.0%), Se-methylseleno-N-acetylgalactosamine (2.9%), and L-selenohomolanthionine (2.5%) [63]. The authors of this study suggested that the high permeability values of MeSeCys and SeMet were due the recognition of these Se compounds by amino acid membrane transporters. Interestingly, their permeability in the presence of methionine was reduced, perhaps due to competition in their recognition by amino acid transporters [63]. After knowing the concentrations in the donor and acceptor wells, it was possible to calculate the percentage of the selenocompound that permeated from the donor well to the acceptor well; the calculated percentages for the Se compounds presented in this work are shown in Table 2. Interestingly, the most permeable Se compounds (**EDA-122** and **EDA-71**) showed permeability percentages of 8.20% and 6.81%, respectively, which were lower than the seleno-amino acids MeSeCys and SeMet reported in [63] but higher than the remaining Se compounds evaluated in that work. The percentage for **EDA-122** was quite high and could be related to the presence of a methylselenol moiety (-SeCH_3_), which is comparable with the terminal ends of MeSeCys and SeMet and could be relevant to membrane permeation because it protects the selenium atom from attacks from thiols and/or free radicals. Additionally, its structure partially resembles the structure of seleno-amino acids.

### 3.4. Metabolic Stability Evaluation 

Another crucial parameter in the field of ADME is the metabolism of the potential drug candidate, which is assessed through the analysis of metabolic stability and through the identification of the most relevant metabolites that appear after the metabolic processing of the administered compound. The metabolites cannot be neglected, because their biological activity and toxicity are frequently crucial for the observed therapeutic or toxicological effects. In metabolic studies, in vitro microsomal models (human, rat, or murine) are widely used [64]. 

Metabolism is defined as the biological modification of drugs and chemicals by enzymatic systems. This leads to the formation of more polar substances that are better excreted from the organism in the urine or bile. The drugs and xenobiotics (non-essential exogenous compounds) undergo biotransformation changes conducted by specific enzymes, which mainly (but not limited to) belong to the cytochrome P450 oxidative system localized in the endoplasmic reticulum of hepatic cells [65]. Therefore, the study of drug metabolic pathways and the potential interactions of a specific tested compound with other drugs is essential to understand the pharmacological activity, toxicity, distribution and excretion of the drug. In this context, the liver is the predominant organ of metabolism for a wide range of endogenous compounds and xenobiotics [66]. 

Traditional in vivo methods used to evaluate the metabolism of drugs are based on small laboratory animals (such as rats, mice, dogs, and rabbits) and determine metabolites after collecting animal plasma and/or urine. Nevertheless, these standard assays are limited by ethical concerns, high costs, the toxicity of the administered drugs, and the small quantity of the isolated metabolites and specious variations. Consequently, there is now a tendency to replace these methods with modern, alternative in vitro methods, such as the use of liver microsomes, microorganisms (fungi), perfused organs, tissues, or cell cultures in biotransformation metabolic studies [29,44,67,68]. 

These alternative methods offer several advantages over animal studies, such as the ability to perform drug screenings for a larger number of strains, relatively lower costs, and the possibility of evaluating regio- and stereo-selective products. Other positive features include alternative methods are the easy detection, isolation, and structure identification of obtained metabolites; the high reproducibility of the experimental procedures; the high reliability and easy validation of the experimental processes [69]. 

Here, we determined the metabolic stability of the oxoselenoester **EDA-71**, firstly using an in silico stability simulation using the MetaSite software and later using human liver microsomes experimentally in vitro. This compound was selected due to its potent and promising biological activity, as reported in previous works [18,19,21,22].

#### 3.4.1. In Silico Metabolic Stability Simulation (MetaSite)

**Figure 3:**
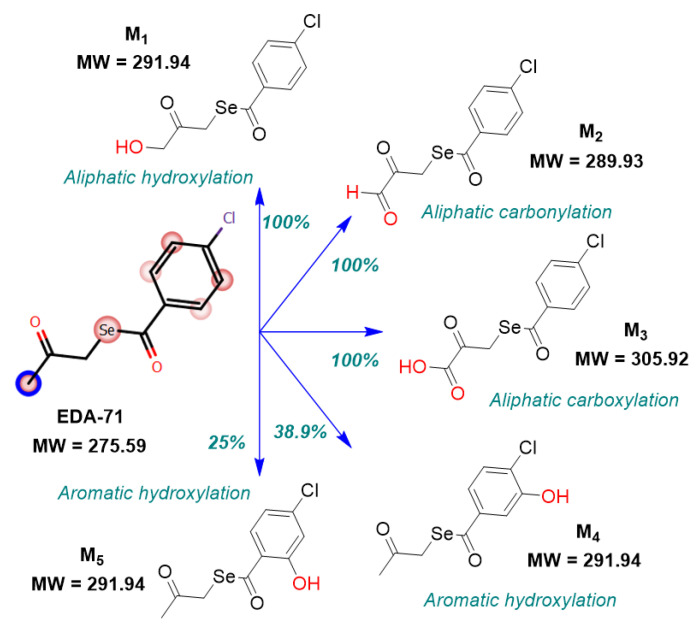
In silico metabolic stability of **EDA-71** selenoester estimated with MetaSite.

The metabolic stability of the Se compound **EDA-71** was determined in silico using MetaSite. Most atoms of its structure were found to be likely to undergo biotransformation, and the structures of the most probable metabolites are shown in Figure 3. 

In this figure, the dark red color of atoms marked with circles indicates a higher probability to be involved in metabolic pathways. The blue circle marks the site of **EDA-71**, which was found to be involved in metabolism with the highest probability (100% score). According to in silico data, the highest probability of metabolism is at the terminal position of selenoester moiety [44].

#### 3.4.2. In Vitro Microsomal Biotransformation Tests

In the next step of the research, a biochemical in vitro method with the use of human liver microsomes (HLMs) was applied to determine the metabolic activity of the compound **EDA-71**. This human liver microsomal model provides all the enzymes required for the phase I metabolism reactions (hydrolysis, oxidation, and reduction). Moreover, it is also possible, in certain conditions following the addition of all necessary cofactors, to conduct metabolism II phase reactions (glutathione transferase and N-acetyltransferase enzymatic reactions). In the drug discovery process, the microsomal is a standard in vitro method for the metabolism evaluation. Additionally, this assay can predict the biotransformation kinetics and intrinsic clearance of new derivatives [69]. Finally, these biotransformation processes can be easily monitored with LC–MS/MS. 

**Figure 4:**
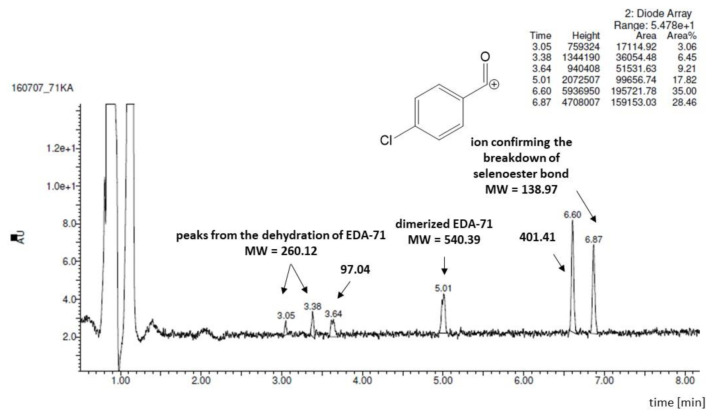
Stability of **EDA-71** in TRIS buffer (pH = 7.4) and LC–MS/MS ionization conditions. Degradation compounds whose mass ranged from *m*/*z*= 97.04 to *m*/*z*= 540.39 were observed, and a maternal compound peak (**EDA-71**, *m*/*z* = 276.60) was not found. Potential degradation compounds observed: dehydration product with *m*/*z* = 260.12; dimer product with *m*/*z* = 540.39; and ion confirming the breakdown of selenoester bond (*m*/*z* = 138.97).

**Figure 5:**
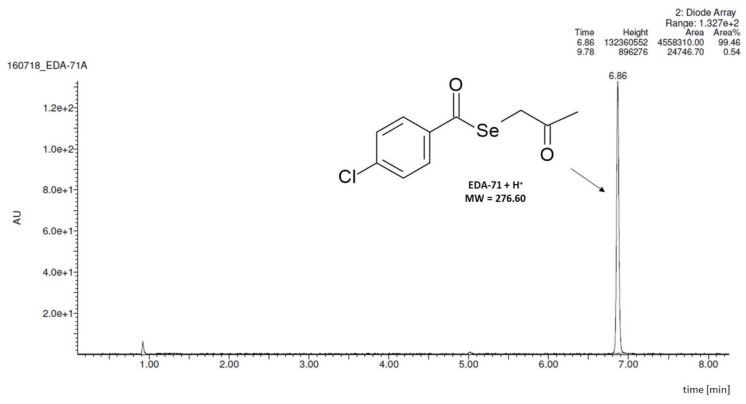
Stability of **EDA-71** during its analysis in organic solvents. The assay confirmed the presence of only one peak, which corresponded to the weight of **EDA-71** (*m*/*z* = 276.60). Diode Array, range: 1.327× 10^2^.

Compound **EDA-71** was studied in this biotransformation assays (Figure 4 and Figure 5) using HLMs, following a previously described procedure [37]. 

The NADPH-regenerating system, which contained a phosphate buffer (pH = 7.4; NADP^+^, glucose-6-phosphate, and glucose-6-phosphate dehydrogenase), was used. The biotransformation process was monitored with the LC–MS/MS method at the end of the procedure at 2 h after the incubation.

Unfortunately, all of the tested selenoesters and selenoanhydride were totally unstable in the TRIS buffer (pH = 7.4, which was used to provide the proper environment of the biological microsomal assay) and under the LC–MS/MS analysis conditions. This experimental fact prevented the detailed analysis of metabolic stability. Interestingly, in this model, all of the tested selenocompounds (**EDA-58**, **EDA-71**, **EDA-119**, **EDA-122**, and **EDA-A6**) were stable in DMSO. Thus, the tested compounds were dissolved in DMSO immediately before the experiment to minimize the degradation risk. The stability of **EDA-71** in the abovementioned conditions is shown in Figure 4 and Figure 5. 

**Figure 6:**
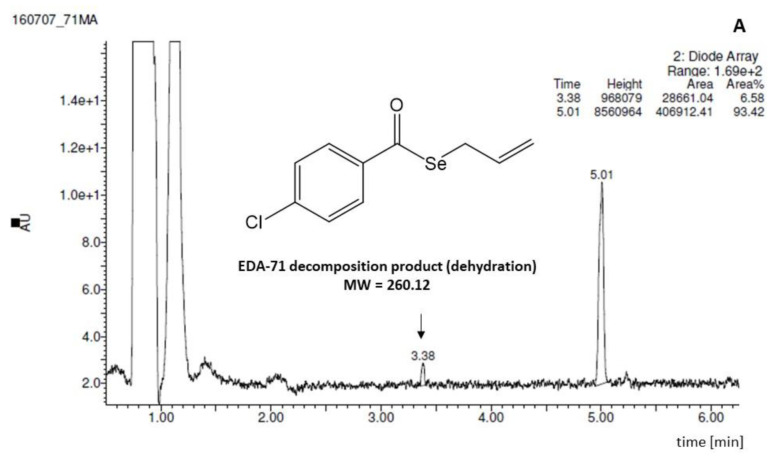

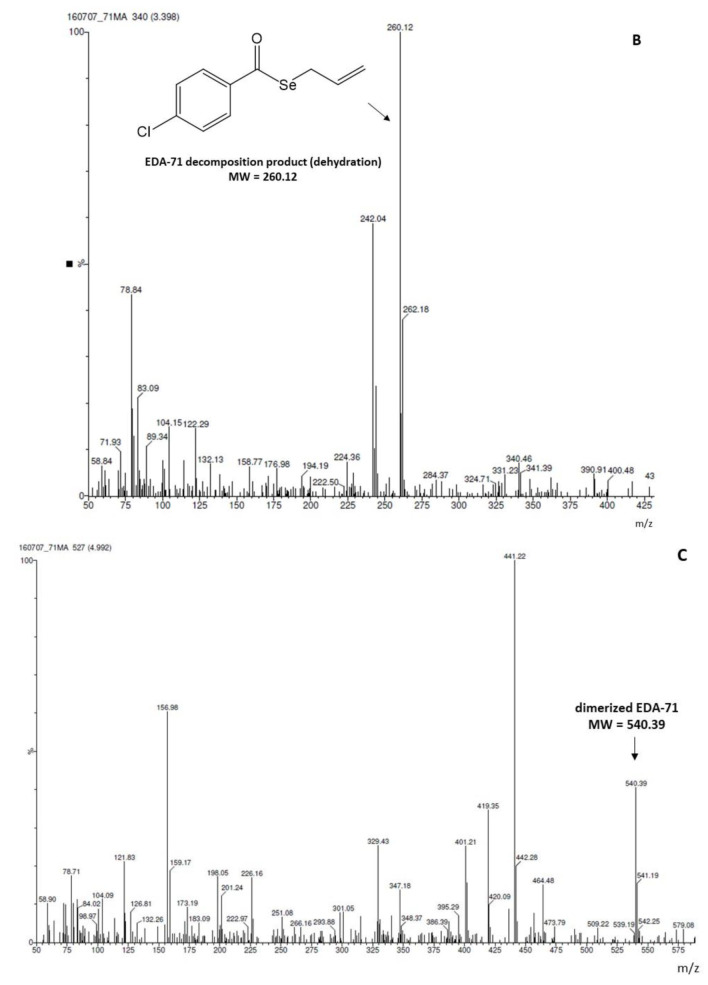
(**A**) The UPLC (ultra-performance liquid chromatography) spectra obtained after the incubation of **EDA-71** with human liver microsomes. Like the control sample with TRIS buffer, it presented a dehydration product (*m*/*z* = 260.12). Diode Array, range: 1.69 × 10^2^. (**B**) MS spectra obtained after the incubation of human liver microsomes with the **EDA-71** selenoester. Like the control sample with TRIS, it showed a dehydration product (*m*/*z* = 260.12). (**C**) MS spectra obtained after the incubation of human liver microsomes with the **EDA-71** selenoester. Like the control sample with TRIS, it showed the **EDA-71** dimer (*m*/*z* = 540.39).

However, the in vitro study (Figure 6A–C) did not confirm the presence of the metabolites estimated with the in silico MetaSite program (Figure 3), though the same dimers and decomposition ions as those observed for the TRIS control (Figure 4) were detected. 

These results showed that the **EDA-71** selenoester may not be stable in the experimental conditions, as it could undergo decomposition or dimerization reactions in the TRIS buffered media (Figure 4 and Figure 6A–C). Specifically, ion fragments with *m*/*z* = 260.12 were present in both the control TRIS probe and the probe after the microsomal biotransformation. This observation may suggest that the dehydration of the tested compound took place in this inorganic environment. Additionally, the ion with *m*/*z* = 138.97 confirmed the breakdown of the carbon–selenium bond (Figure 4 and Figure 6A–C).

As mentioned above, it was shown in a previous work [20] that the hydrolysis of selenoanhydride releases selenium anions or reactive selenium species that can quickly react with thiols (such as glutathione or hydrogen sulfide), reactive oxygen species (such as hydrogen peroxide), and free radicals (like superoxide) naturally present in human cells [20]. Because these reactive selenium species quickly react in cellular environments, they can be elusive and difficult to identify.

The authors of another relevant study [70] watered a selenium-accumulator plant (*Brassica oleracea* L. var. *sabellica* L.) with water containing Se compounds (mainly selenoesters) in solution. Later, the metabolites generated by this plant were studied. Although the metabolism of the studied plant was not comparable with the metabolism in the microsomes, the results revealed a very complex mixture of selenium metabolites, including complex sulfur–selenium species [70], thus supporting the hypothesis of their generation in human cells from the hydrolysis of these compounds (as it has been suggested in previous works [18,20]).

This observed experimental lack of metabolic stability may not be completely negative for the pharmaceutical profile evaluation of the compound, as it can be considered a line of evidence that proves the release of selenium anions or of reactive selenium species that are then available to interact with cellular targets. This release could be the mechanism of action responsible of the biological activities of this derivative described so far [18]. Therefore, for these compounds used as prodrugs, the ease of this release can be considered to be more of a strength than a weakness. 

Interestingly, this breakdown was not predicted in silico by MetaSite software. The reason for this lack of prediction could be the same as that described in the discussion of the differences between the experimental compound solubility data and those determined with different in silico programs (Chemicalize, Osiris, and SwissADME): selenium is an infrequent element in organic compounds, so predictions for compounds that include this element in their chemical structures can have less reliable predictions when their properties are simulated by the use of these programs. Here, we confirmed this deviation effect in the assessment of solubility and metabolic stability using three bioinformatic tools and one commercial program, respectively. 

### 3.5. Evaluation of the Mutagenicity through the Ames Test

Before their approval to be sold in the market to treat specific diseases, every drug must undergo a series of studies to determine its structure, physicochemical properties, mechanism of action, and side effects [71]. A further requirement for newly synthesized compounds is the precise estimation of their safety profiles: only non-toxic and non-mutagenic derivatives have a chance to reach the further steps of the experiments. In this context, it is extremely important to determine the mutagenic potential of a drug candidate, because the results of mutagenicity tests often determine whether a tested compound can be introduced to clinical trials. As mentioned in the metabolic stability section, the evaluation of drugs with animals creates serious ethical dilemmas. Although experiments on animals are regulated by law, frequently it is not possible to eliminate the suffering of laboratory animals [72]. Additionally, in vitro assays reduce the amount of animals used during research procedures according to the replacement, reduction, and refinement approach (3Rs approach), which was first promulgated by Russel and Burch in 1959 [73]. Therefore, an excellent solution, which minimizes the usage of laboratory animals in the early stages of research and enables the study of a greater number of compounds, may be the use of alternative bacterial tests that easily enable the estimation of the mutagenic potential of tested compounds. Nowadays, the in vitro Ames alternative bacterial test using the *Salmonella* Typhimurium TA-100 strain is considered the gold standard of in vitro gene mutation genotoxicity testing [29,46,74]. 

Some substances or their metabolites possess the ability to damage the genetic material of cells. There are various mechanisms of action of these compounds, and one of the most common is the formation of adducts with DNA. As a result, the alkylation of the purine and pyrimidine bases occurs. Very often, a genotoxic substance also has carcinogenic potential. It has been proven that 90% of carcinogenic compounds can cause mutations in the bacterial strains used in the most widespread microbiological mutagenicity tests. All carcinogenic compounds are mutagens, but not every mutagen is responsible for the appearance of a carcinogenic effect. It has been shown that the sensitivity of DNA bases to mutagens increases during the course of the replication process in cell division. Therefore, the probability of DNA damage is dependent on exposure to mutagens, as well as on the frequency of cell divisions [75].

By definition, a mutation is a permanent and hereditary change in the quality and quantity of information contained in the genetic material of cells or organisms, which can occur in somatic and/or germ cells and can alter the structure and functions of cell proteins and enzymes. The most common causes of mutations are physical factors (e.g., UV radiation) and chemical exposure. The most popular tools for detecting point gene mutations are simple bacterial tests. Due to the high frequency of replication, bacteria play key roles in the mechanism of test function. If an assay is properly performed, it provides reliable and reproducible results that can be easily read [76]. Among these bacterial tests, the Ames test is recognized as one of the most commonly performed genotoxicity assays by industrial organizations [47,77].

Here, the mutagenicity of the selenocompounds was evaluated using both in silico methods (simulation in OSIRIS software) and in vitro methods (microplate mutagenicity Ames assay).

#### 3.5.1. Safety Profile In Silico Results

Mutagenicity and other parameters connected with the safety of the potential drug candidates were evaluated during in silico analysis with the use of the free OSIRIS program. This software enables the preliminary description of the mutagenic and tumorigenic potential of tested compounds. Additionally, OSIRIS was used to evaluate whether compounds caused reproductive effects or were irritant compounds. 

According to this OSIRIS in silico tool, 12 of the tested compounds (**EDA-26**, **EDA-46**, **EDA-53**, **EDA-58**, **EDA-71**, **EDA-73**, **EDA-74**, **EDA-93**, **EDA-109**, **EDA-117**, **EDA-120**, and **EDA-A6**) exhibited in silico tumorigenic effects. None of the 15 tested compounds showed mutagenicity, irritant properties, or influence on the reproductive effect. 

#### 3.5.2. Safety Profile In Vitro Results in Ames Microplate Mutagenicity Assay 

**Figure 7:**
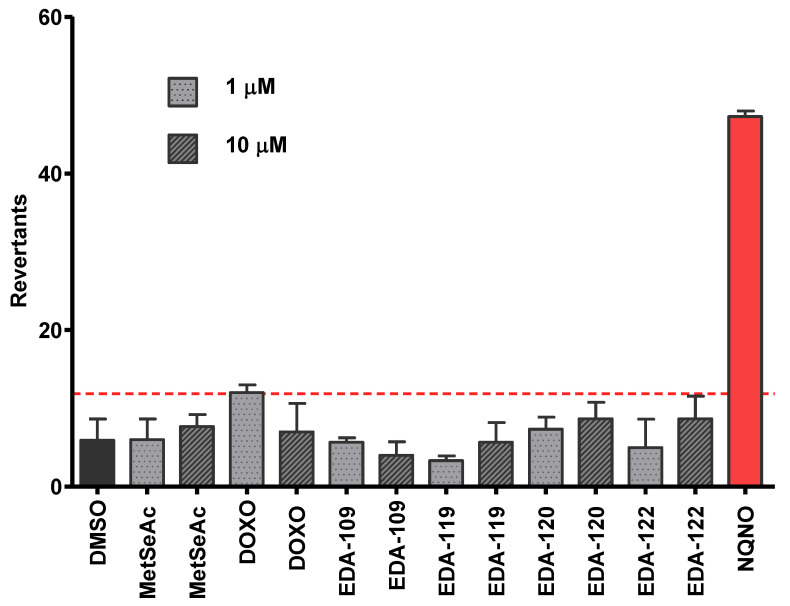
Safety profile (384-well microfluctuation Ames test) for the selenoesters with very high cytostatic activity, DMSO—solvent control. All tested compounds and references were evaluated at 1 and 10 µM concentrations, except for NQNO (0,5 µM); **----** baseline defining the mutagenicity threshold (over this line).

**Figure 8:**
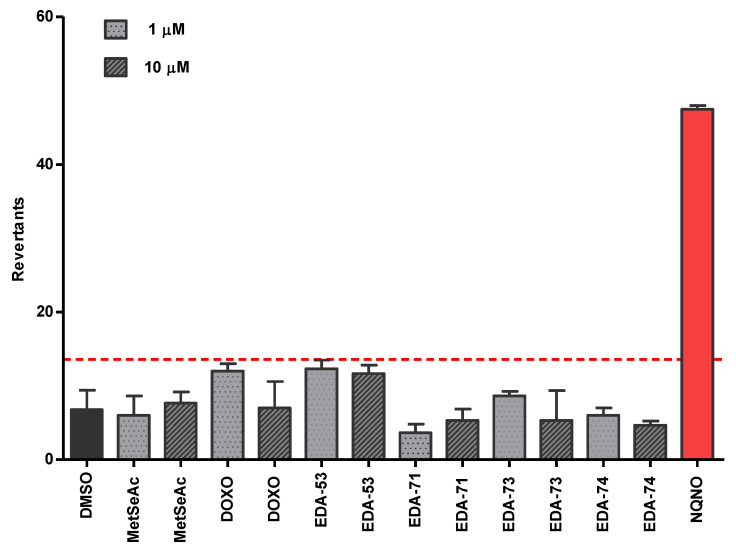
Safety profile (384-well microfluctuation Ames test) for the selenoesters with high cytostatic activity. DMSO—solvent control. All tested compounds and references were evaluated at 1 and 10 µM concentrations, except for NQNO (0,5 µM); **----** baseline defining the mutagenicity threshold (over this line).

**Figure 9:**
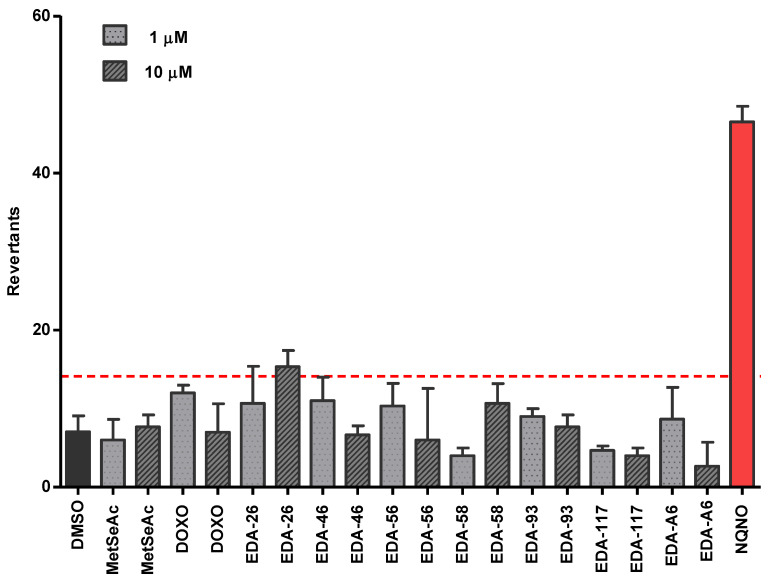
Safety profile (384-well microfluctuation Ames test) for the selenoesters with significant cytostatic activity. DMSO—solvent control. All tested compounds and references were evaluated at 1 and 10 µM concentrations, except for NQNO (0,5 µM); ---- baseline defining the mutagenicity threshold (over this line).

In the next step of the studies, the in vitro safety profiles of the tested selenocompounds were evaluated. All compounds were tested with the 384-well plate microfluctuation Ames test. The mutagenic index (MI) was used as the measure of mutagenicity [29,46]. The results of the Ames tests, which determined the mutagenic potential of the tested compounds, are shown in Figure 7, Figure 8 and Figure 9 and collected in Table 3, Table 4 and Table 5.

None of the four tested selenoesters with very high cytostatic activity were mutagenic. However, in the case of the **EDA-120**, the binomial B-value (1.00) slightly exceeded the threshold (0.99) at the two evaluated concentrations. Nevertheless, this compound was defined as non-mutagenic due to the correct fold increase over the baseline, which was below 2.0 in both cases. A similar situation was observed in the standard cytostatic compound (doxorubicin) at the 1 µM concentration. Interestingly, for the **EDA-109** derivative, cytotoxic activity was observed at the highest concentration (10 µM) (Figure 7 and Table 3).

None of the four selenoesters with high cytostatic activity were found to be mutagenic. The binomial B-value (1.00) slightly exceeded the threshold (0.99) in the case of the selenocompound **EDA-53** at a concentration of 1 µM (Figure 8 and Table 4). However, like compound **EDA-120**, it was considered non-mutagenic because it did not pass the baseline threshold.

After testing the seven selenocompounds with significant cytostatic activity (Figure 9 and Table 5), it turned out that the **EDA-26** derivative may have had mutagenic potential at the highest tested concentration, as indicated by its mutagenic index value (MI ≥ 2) and binomial B-value (≥0.99). 

Besides **EDA-26**, none of the remaining six selenoesters (Figure 9 and Table 5) with significant cytostatic activity displayed mutagenic effects in this assay. It is necessary to point out that the binomial B-values for compounds **EDA-46** (at 1 µM) and **EDA-58** (at 10 µM) slightly exceeded (1.00) the 0.99 threshold, below which mutations are considered to be spontaneous. This was the same as was observed in previous compounds (**EDA-53** and **EDA-120**), which were not considered mutagenic because the number of revertants increased by less than 2-fold over the baseline. It is worth noting that the selenoanhydride **EDA-A6** could have been cytotoxic for *Salmonella* Typhimurium at the 10 µM concentration, so this substance may have interfered with the Ames test results at the higher concentrations.

The results of the HTS-adapted microplate Ames test indicated that all of the tested selenoesters and selenoanhydride derivatives were safe and devoid of mutagenic activity, with the exception of the compound **EDA-26**, which slightly exceeded the baseline at the 10 µM concentration. In this case, the results were in accordance with those obtained with the in silico method (OSIRIS program), where none of tested selenocompounds were shown to exhibit mutagenicity.

#### 3.5.3. Reference Experiment Results—The Impact of Tested Selenocompounds on the *Salmonella* Typhimurium Growth

Some scientific reports have indicated strong antimicrobial activity against “ESKAPE-pathogens” of certain groups of organic selenium compounds, namely selenazolinium salts and certain aromatic selenocyanates [38,46]. This ESKAPE acronym includes the most troublesome bacteria that are resistant to conventionally used antibiotics: vancomycin-resistant *enterococci* (VRE), methicillin-resistant *Staphylococcus aureus* (MRSA), *Klebsiella pneumoniae*, *Acinetobacter baumannii*, *Pseudomonas aeruginosa*, and extended-spectrum *β*-lactamase (ESBL)-producing or carbapenem-resistant species of the family *Enterobacteriaceae* (CRE) [46]. 

**Figure 10:**
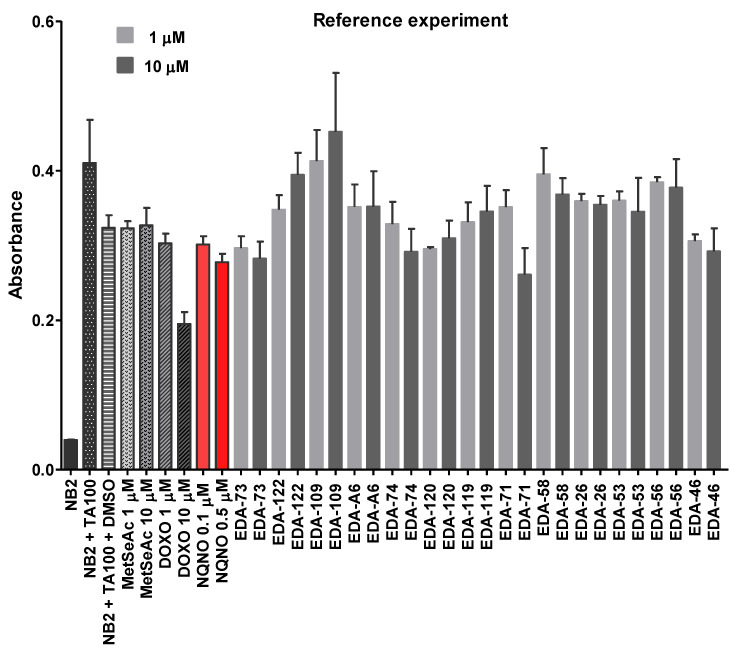
Reference experiment results for organic selenocompounds in two concentrations: 1 µM (marked with light grey) and 10 µM (marked with dark grey); NB2—nutrient broth no. 2 without bacteria (background control). NB2 + TA100—control with *Salmonella* Typhimurium TA100 strain alone; NB2 + TA100 + DMSO—solvent control; MetSeAc—methylseleninic acid (anticancer selenocompound); DOXO—doxorubicin (standard cytostatic compound).

Therefore, a reference experiment with 20 h of incubation at constant temperature conditions (37 °C) was performed in order to determine the effect of the tested compounds on the growth of the *Salmonella* Typhimurium used in the Ames test. The results of this experiment showed no inhibitory effects on the growth and development of this bacteria (Figure 10). Therefore, this method could be successfully applied to determine the safety profile of the tested derivatives. In addition, this test was performed to confirm or exclude the results from the Ames test, especially the values that indicated the possibility of the cytotoxic potential of several compounds (for which the binomial B-value was significantly smaller (B ≤ 0.01) than the mean number of spontaneous revertants (values labelled as “cytotoxic effect”)). 

However, the results of the reference test for the **EDA-109** selenoester indicated some contradictions with the result of the Ames test. It is noteworthy that this compound showed a stimulating effect on the growth of *Salmonella* Typhimurium, especially at a higher concentration (10 μM) after 20 h of incubation in the reference experiment. On the other hand, the results of the Ames test presented values that were significantly smaller (B ≤ 0.01) than the mean number of spontaneous revertants, labelled as C “cytotoxic effect”. In line with these results showing a lack of activity at the 10 µM concentration in the reference test, the derivatives of these compounds were evaluated in a previous work [78] as antibacterial agents against different bacterial strains of *Salmonella* Typhimurium, *Staphylococcus aureus* and *Pseudomonas aeruginosa*, and it was found that the MIC values in *Salmonella* Typhimurium reached 50–100 µM depending on the strain and of the compound.

However, there was a significant difference in both experimental procedures related to incubation time, which was 72 h in the Ames test. This incubation time allowed for the more accurate determination of the long-term effects of the tested compound on the bacterial culture than in the reference test, which used 20 h of incubation. Therefore, the observed differences between the two tests were most likely related to the incubation time. In addition, it has been confirmed that selenocompounds such as sodium selenite [79] and selenomethionine [80], in non-toxic concentrations, may have stimulating effects on cell growth in the initial phase [79,80]. 

## 4. Conclusions

The pharmaceutical profiles and safety of 15 selenocompounds with noteworthy biological activity reported in previous works [18,19,20,21,22] were evaluated in this work. These Se compounds comprised 14 selenoester and a cyclic selenoanhydride, the phthalic selenoanhydride **EDA-A6**. 

The 12 selenocompounds were experimentally found to have low solubility values (according to the Biopharmaceutical Classification System) in the range from 0.001 to 1 g/L. The experimental chemical stability of 11 of these 15 Se compounds was assessed in two different environments and we found that all the tested compounds were unstable in alkaline conditions, and some of them were also unstable in acidic conditions. Similarly, the compound evaluated in experimental metabolic assays (**EDA-71**) was unstable in the TRIS buffer used in human liver microsomes (HLMs), as peaks related to hydrolysis and the dimerization products were found in both the HLM and control experiments with this buffer. Concerning their permeability, five Se compounds were tested with PAMPA assay and capillary electrophoresis, and they showed high permeability values. Remarkably, three of them were more permeable than the reference drug, caffeine. Finally, the mutagenicity of the 15 selenocompounds was evaluated with an Ames microplate test, and we found that, with the exception of **EDA-26** at the 10 µM concentration, they were non-mutagenic. 

These compounds have been used as prodrugs with the aim to deliver selenium to target cells, where they can release selenium anions or reactive selenium species that are ultimately responsible for the observed biological activities, according to previous works. Therefore, their lack of stability in chemical and metabolic assays may not be a weakness, because it may support the hypothesis of their design: their instability may enable the desired release and confirm the potential use of these derivatives as prodrugs. This hypothesis is supported by the high observed permeability of the compounds, which may make them suitable as vehicles for the delivery of selenium into cells. 

Interestingly, when the experimental in vitro results of the solubility and metabolic stability of the compounds were compared with in silico predictions obtained using specialized software (Chemicalize, SwissADME, and OSIRIS for the solubility; MetaSite for the metabolism), very significant differences were observed. The reason for these divergences may be that the tested compounds contain selenium atoms, and it is possible that this element, scarcely found in most organic compounds, is not correctly parametrized in these in silico software programs. 

In conclusion, we conducted the preliminary determination of the pharmaceutical profiles and safety of a promising series of selenium derivatives. The results showed that the evaluated compounds have high permeability, are non-mutagenic, and may easily undergo reactions of hydrolysis inside cells. This supports the idea that they may behave as prodrugs that release the species ultimately responsible for the observed biological activities. However, more research, including the evaluation of more compounds and the use of more assays, is needed to determine aspects of importance, such as the nature and the activity of the produced metabolites. In any case, according to our knowledge, this is the first experimental ADME study of this type of selenocompounds, and these results are of interest for future, more in-depth studies; so far, only two ADME studies in silico have been performed [81,82].

## Figures and Tables

**Table 1:** Aqueous solubility determined using computational and experimental methods, as well as percentage that the predicted in silico solubility predicted in respect to the aqueous experimental solubility. In experimental values, green denotes almost insoluble compounds and blue denotes those compounds with solubility of more than 1 g/L.

Tested Compound	Experimental Aqueous Solubility		In Silico Calculated Aqueous Solubility			% That the In Silico Value Supposes in Respect to the Aqueous Experimental Solubility		
	[g/L]	logS_m_	Chemic logS_m_	SwissA logS_m_^1^	OSIR logS_m_	Chemic	SwissA	OSIR
**EDA-26**	0.380	−2.96	−4.02	−5.20	−5.06	8.71%	0.58%	0.79%
**EDA-71**	0.132	−3.32	−4.85	−5.53	−5.96	2.95%	0.62%	0.23%
**EDA-73**	**~0.001**	**~−5.50**	−5.82	−5.59	−6.63	47.9%	81.3%	7.41%
**EDA-74**	0.019	−4.26	−5.10	−6.03	−5.97	14.5%	1.70%	1.95%
**EDA-93**	0.022	−4.16	−5.95	−6.40	−6.30	1.62%	0.58%	0.72%
**EDA-109**	0.028	−4.06	−3.53	−6.13	−6.16	339%	0.85%	0.79%
**EDA-117**	**1.028**	**−2.37**	−3.81	−4.21	−4.79	3.63%	1.45%	0.38%
**EDA-119**	0.137	−3.37	−2.82	−5.88	−5.41	355%	0.31%	0.91%
**EDA-120**	0.026	−4.09	−3.53	−6.13	−6.16	363%	0.91%	0.85%
**EDA-122**	0.031	−4.02	−3.76	−6.35	−6.28	182%	0.47%	0.55%
**EDA-A6**	0.758	−2.44	−4.29	−4.91	−5.68	1.41%	0.34%	0.06%

^1^ Abbreviations: LogSm—calculated based on solubility S_m_ (mol/L); Chemic—Chemicalize; SwissA—SwissADME; OSIR—OSIRIS.

**Table 2:** Results of the experimental in vitro permeability test (PAMPA) for selected organic selenocompounds, together with references with high or low permeability (caffeine or norfloxacin). The percentage of compound or reference that crossed from the donor well to the acceptor well (% Perm.) is shown in parentheses.

Compound	In Vitro Permeability [cm/s] (% Perm.)	Compound	In Vitro Permeability [cm/s] (% Perm.)	Reference	In Vitro Permeability [cm/s] (% Perm.)
**EDA-58**	2.220 × 10^−6^ (3.95%)	**EDA-122**	4.668 × 10^−6^ (8.20%)	**Caffeine**	3.420 × 10^−6^ (6.05%)
**EDA-71**	3.858 × 10^−6^ (6.81%)	**EDA-A6**	3.438 × 10^−6^ (6.08%)	**Norfloxacin**	0.9488 × 10^−7^ (1.70%)
**EDA-119**	3.271 × 10^−6^ (5.79%)				

Compounds in blue showed a higher permeability than caffeine.

**Table 3:** Mutagenic index (MI) and other values for methylseleninic acid (reference Se compound), doxorubicin (reference chemotherapeutic), and tested compounds with high cytostatic activity.

Compound	Mutagenicity at 1 µM Concentration				Mutagenicity at 10 µM Concentration			
	MI ^1^	Fold Increase over Baseline	B ^2^	SD	MI	Fold Increase over Baseline	B	SD
**MetSeAc**	0.90	0.75	0.37	2.65	0.96	0.74	0.47	1.53
**DOXO**	1.64	1.31	**1.00**	1.00	0.95	0.77	0.47	3.61
**EDA-109**	0.96	0.55	0.07	0.58	0.67	0.39	**0.003 C**	1.73
**EDA-119**	0.56	0.54	0.24	0.58	0.96	0.92	0.90	2.52
**EDA-120**	1.24	1.23	**1.00**	1.53	1.46	1.45	**1.00**	2.08
**EDA-122**	0.84	0.63	0.14	3.61	1.46	1.08	0.94	2.89

^1^ MI (mutagenic index) = mean revertant value for the tested compound/mean revertant value for the blind probe with DMSO; compound was considered to be mutagenic if MI ≥ 2; baseline = mean + 1 SD. ^2^ B: the binomial B value indicates the probability that spontaneous mutation events alone produce at most n = total number of yellow wells at a given concentration of the test sample (3 × 48 wells); a binomial B-value ≥ 0.99 (bolded) indicates that chances are ≤1% that this result occurred due to spontaneous mutation; data points that are significantly smaller (B ≤ 0.01 (bolded)) than the mean number of spontaneous revertants are labelled C for “cytotoxic effect”. A compound is considered to be mutagenic when its MI and B values show fold increases ≥ 2 and ≥ 0.99, respectively.

**Table 4:** Mutagenic index (MI) and other values for methylseleninic acid (reference Se compound), doxorubicin (reference anticancer agent), and tested derivatives with high cytostatic activity.

Compound	Mutagenicity at 1 µM Concentration				Mutagenicity at 10 µM Concentration			
	MI	Fold Increase over Baseline	B	SD	MI	Fold Increase over Baseline	B	SD
**MetSeAc**	0.90	0.75	0.37	2.65	0.96	0.74	0.47	1.53
**DOXO**	1.64	1.31	**1.00**	1.00	0.95	0.77	0.46	3.61
**EDA-53**	1.82	1.10	**1.00**	12.33	1.72	1.04	0.98	11.67
**EDA-71**	0.54	0.60	0.34	1.15	0.79	0.89	0.98	1.53
**EDA-73**	1.28	0.84	0.72	0.58	0.79	0.52	0.04	4.04
**EDA-74**	0.88	0.98	0.94	1.00	0.69	0.76	0.68	0.58

Bolded B values indicate risk of cytotoxic or mutagenic effects (see footnote of Table 3 for full description).

**Table 5:** Mutagenic index (MI) and other values for methylseleninic acid (reference Se compound), doxorubicin (reference anticancer agent), and tested compounds with significant cytostatic activity.

Compound	Mutagenicity at 1 µM Concentration				Mutagenicity at 10 µM Concentration			
	MI	Fold Increase over Baseline	B	SD	MI	Fold Increase over Baseline	B	SD
**MetSeAc**	0.90	0.75	0.37	2.65	0.96	0.74	0.47	1.53
**DOXO**	1.64	1.31	**1.00**	1.00	0.95	0.77	0.46	3.61
**EDA-26**	1.51	0.96	0.92	4.73	**2.16**	1.37	**1.00**	2.08
**EDA-46**	1.56	1.65	**1.00**	3.00	0.94	1.00	0.88	1.15
**EDA-56**	1.46	0.93	0.88	2.89	0.85	0.90	0.75	6.56
**EDA-58**	0.57	0.67	0.80	1.00	1.51	1.78	**1.00**	2.52
**EDA-93**	1.27	1.02	0.96	1.00	1.08	0.87	0.80	1.53
**EDA-117**	0.66	0.49	0.36	0.58	0.57	0.42	0.18	1.00
**EDA-A6**	1.23	1.08	0.94	4.04	0.38	0.33	**0.001 C**	3.06

Bolded B values indicate risk of cytotoxic or mutagenic effects (see footnote of Table 3 for full description). Bolded MI value denotes mutagenicity.

## Data Availability

The data presented in this study are available in this article.

## Supplementary Materials

A detailed description of the material and methods is available online at https://www.mdpi.com/article/10.3390/pharmaceutics14020367/s1.

## References

[1] Rayman M.P. (2020). Selenium intake, status, and health: A complex relationship. Hormones.

[2] Nogueira C.W., Rocha J.B. (2011). Toxicology and pharmacology of selenium: Emphasis on synthetic organoselenium compounds. Arch. Toxicol..

[3] Wang X., Yang Y., Zhang H., Liu J. (2017). Safety Assessment and Comparison of Sodium Selenite and Bioselenium Obtained from Yeast in Mice. Biomed. Res. Int..

[4] Radomska D., Czarnomysy R., Radomski D., Bielawska A., Bielawski K. (2021). Selenium as a Bioactive Micronutrient in the Human Diet and Its Cancer Chemopreventive Activity. Nutrients.

[5] Rayman M.P. (2000). The importance of selenium to human health. Lancet.

[6] Peyroche G., Saveanu C., Dauplais M., Lazard M., Beuneu F., Decourty L., Malabat C., Jacquier A., Blanquet S., Plateau P. (2012). Sodium selenide toxicity is mediated by O2-dependent DNA breaks. PLoS ONE.

[7] Zhang G., Nitteranon V., Guo S., Qiu P., Wu X., Li F., Xiao H., Hu Q., Parkin K.L. (2013). Organoselenium compounds modulate extracellular redox by induction of extracellular cysteine and cell surface thioredoxin reductase. Chem. Res. Toxicol..

[8] Brozmanová J., Mániková D., Vlčková V., Chovanec M. (2010). Selenium: A double-edged sword for defense and offence in cancer. Arch. Toxicol..

[9] Lee K.H., Jeong D. (2012). Bimodal actions of selenium essential for antioxidant and toxic pro-oxidant activities: The selenium paradox (Review). Mol. Med. Rep..

[10] Weekley C.M., Harris H.H. (2013). Which form is that? The importance of selenium speciation and metabolism in the prevention and treatment of disease. Chem. Soc. Rev..

[11] Jaworska K., Gupta S., Durda K., Muszyńska M., Sukiennicki G., Jaworowska E., Grodzki T., Sulikowski M., Waloszczyk P., Wójcik J. (2013). A low selenium level is associated with lung and laryngeal cancers. PLoS ONE.

[12] Bartolini D., Sancineto L., Fabro de Bem A., Tew K.D., Santi C., Radi R., Toquato P., Galli F. (2017). Selenocompounds in Cancer Therapy: An Overview. Adv. Cancer Res..

[13] Radomska D., Czarnomysy R., Radomski D., Bielawski K. (2021). Selenium Compounds as Novel Potential Anticancer Agents. Int. J. Mol. Sci..

[14] Ali W., Álvarez-Pérez M., Marć M.A., Salardón-Jiménez N., Handzlik J., Domínguez-Álvarez E. (2018). The Anticancer and Chemopreventive Activity of Selenocyanate-Containing Compounds. Curr. Pharmacol. Rep..

[15] Chuai H., Zhang S.Q., Bai H., Li J., Wang Y., Sun J., Wen E., Zhang J., Xin M.K. (2021). Small molecule selenium-containing compounds: Recent development and therapeutic applications. Eur. J. Med. Chem..

[16] Fernandes A.P., Gandin V. (2015). Selenium compounds as therapeutic agents in cancer. Biochim. Biophys. Acta.

[17] Kim S.J., Choi M.C., Park J.M., Chung A.S. (2021). Antitumor Effects of Selenium. Int. J. Mol. Sci..

[18] Domínguez-Álvarez E., Plano D., Font M., Calvo A., Prior C., Jacob C., Palop J.A., Sanmartín C. (2014). Synthesis and antiproliferative activity of novel selenoester derivatives. Eur. J. Med. Chem..

[19] Gajdács M., Spengler G., Sanmartín C., Marć M.A., Handzlik J., Domínguez-Álvarez E. (2017). Selenoesters and selenoanhydrides as novel multidrug resistance reversing agents: A confirmation study in a colon cancer MDR cell line. Bioorg. Med. Chem. Lett..

[20] Kharma A., Misak A., Marian Grman G., Brezova V., Kurakova L., Baráth P., Jacob C., Chovanec M., Ondrias K., Domínguez-Álvarez E. (2019). Release of reactive selenium species from phthalic selenoanhydride in the presence of hydrogen sulfide and glutathione with implications for cancer research. New J. Chem..

[21] Spengler G., Kincses A., Mosolygó T., Marć M.A., Nové M., Gajdács M., Sanmartín C., McNeil H.E., Blair J.M.A., Domínguez-Álvarez E. (2019). Antiviral, Antimicrobial and Antibiofilm Activity of Selenoesters and Selenoanhydrides. Molecules.

[22] Mosolygó T., Kincses A., Csonka A., Tönki Á.S., Witek K., Sanmartín C., Marć M.A., Handzlik J., Kieć-Kononowicz K., Domínguez-Álvarez E. (2019). Selenocompounds as Novel Antibacterial Agents and Bacterial Efflux Pump Inhibitors. Molecules.

[23] Di L., Kerns E.H., Carter G. (2009). Drug-like property concepts in pharmaceutical design. Curr. Pharm. Des..

[24] Kerns E.H., Di L. (2008). Drug-like Properties: Concepts, Structure, Design and Methods.

[25] Kong W.M., Chik Z., Mohamed Z., Alshawsh M.A. (2017). Physicochemical Characterization of Mitragyna speciosa Alkaloid Extract and Mitragynine Using *In Vitro* High Throughput Assays. Comb. Chem. High. Throughput Screen.

[26] Alam S., Khan F. (2019). 3D-QSAR, Docking, ADME/Tox studies on Flavone analogs reveal anticancer activity through Tankyrase inhibition. Sci. Rep..

[27] DeMarini D.M., Shelton M.L., Warren S.H., Ross T.M., Shim J.Y., Richard A.M., Pegram R.A. (1997). Glutathione S-transferase-mediated induction of GC-->AT transitions by halomethanes in Salmonella. Environ. Mol. Mutagen..

[28] DeMarini D.M., Abu-Shakra A., Felton C.F., Patterson K.S., Shelton M.L. (1995). Mutation spectra in salmonella of chlorinated, chloraminated, or ozonated drinking water extracts: Comparison to MX. Environ. Mol. Mutagen..

[29] Marć M.A., Domínguez-Álvarez E., Słoczyńska K., Żmudzki P., Chłoń-Rzepa G., Pękala E. (2018). In Vitro Biotransformation, Safety, and Chemopreventive Action of Novel 8-Methoxy-Purine-2,6-Dione Derivatives. Appl. Biochem. Biotechnol..

[30] Mortelmans K., Zeiger E. (2000). The Ames Salmonella/microsome mutagenicity assay. Mutat. Res..

[31] Zeiger E. (2013). Bacterial mutation assays. Methods Mol. Biol..

[32] Ames B.N., Lee F.D., Durston W.E. (1973). An improved bacterial test system for the detection and classification of mutagens and carcinogens. Proc. Natl. Acad. Sci. USA.

[33] Southan C., Stracz A. (2013). Extracting and connecting chemical structures from text sources using chemicalize.org. J. Cheminform..

[34] Daina A., Michielin O., Zoete V. (2017). SwissADME: A free web tool to evaluate pharmacokinetics, drug-likeness and medicinal chemistry friendliness of small molecules. Sci. Rep..

[35] Sander T., Freyss J., von Korff M., Reich J.R., Rufener C. (2009). OSIRIS, an entirely in-house developed drug discovery informatics system. J. Chem. Inf. Model.

[36] Vollmann K., Qurishi R., Hockemeyer J., Müller C.E. (2008). Synthesis and properties of a new water-soluble prodrug of the adenosine A 2A receptor antagonist MSX-2. Molecules.

[37] Żesławska E., Kucwaj-Brysz K., Kincses A., Spengler G., Szymańska E., Czopek A., Marć M.A., Kaczor A., Nitek W., Domínguez-Álvarez E. (2021). An insight into the structure of 5-spiro aromatic derivatives of imidazolidine-2,4-dione, a new group of very potent inhibitors of tumor multidrug resistance in T-lymphoma cells. Bioorg. Chem..

[38] Nasim M.J., Witek K., Kincses A., Abdin A.Y., Żesławska E., Marć M.A., Gajdács M., Spengler G., Nitek W., Latacz G. (2019). Pronounced activity of aromatic selenocyanates against multidrug resistant ESKAPE bacteria. New J. Chem..

[39] Chen X., Murawski A., Patel K., Crespi C.L., Balimane P.V. (2008). A novel design of artificial membrane for improving the PAMPA model. Pharm. Res..

[40] Bujard A., Voirol H., Carrupt P.A., Schappler J. (2015). Modification of a PAMPA model to predict passive gastrointestinal absorption and plasma protein binding. Eur. J. Pharm. Sci..

[41] Latacz G., Lubelska A., Jastrzębska-Więsek M., Partyka A., Sobiło A., Olejarz A., Kucwaj-Brysz K., Satała G., Bojarski A.J., Wesołowska A. (2017). In the search for a lead structure among series of potent and selective hydantoin 5-HT7 R agents: The drug-likeness in vitro study. Chem. Biol. Drug Res..

[42] Łażewska D., Więcek M., Ner J., Kamińska K., Kottke T., Schwed J.S., Zygmunt M., Karcz T., Olejarz A., Kuder K. (2014). Aryl -1,2,5-triazine derivatives as histaminę H4 receptor ligands. Eur. J. Med. Chem..

[43] Kucwaj-Brysz K., Warszycki D., Podlewska S., Witek J., Witek K., González Izquierdo A., Satała G., Loza M.I., Lubelska A., Latacz G. (2016). Rational design in search for 5-phenylhydantoin selective 5-HT7R antagonists. Molecular modeling, synthesis and biological evaluation. Eur. J. Med. Chem..

[44] Kamiński K., Zagaja M., Łuszczki J.J., Rapacz A., Andres-Mach M., Latacz G., Kieć-Kononowicz K. (2015). Design, Synthesis, and Anticonvulsant Activity of New Hybrid Compounds Derived from 2-(2,5-Dioxopyrrolidin-1-yl)propanamides and 2-(2,5-Dioxopyrrolidin-1-yl)butanamides. J. Med. Chem..

[45] Sadek B., Saad A., Latacz G., Kuder K., Olejarz A., Karcz T., Stark H., Kieć-Kononowicz K. (2016). Non-imidazole-based histamine H3 receptor antagonists with anticonvulsant activity in different seizure models in male adult rats. Drug Des. Dev. Ther..

[46] Witek K., Nasim M.J., Bischoff M., Gaupp R., Arsenyan P., Vasiljeva J., Marć M.A., Olejarz A., Latacz G., Kieć-Kononowicz K. (2017). Selenazolinium Salts as “Small Molecule Catalysts” with High Potency against ESKAPE Bacterial Pathogens. Molecules.

[47] Flückiger-Isler S., Baumeister M., Braun K., Gervais V., Hasler-Nguyen H., Reimann R., van Gompel J., Wunderlich H.-G., Engelhardt G. (2004). Assessment of the performance of the Ames IITM assay: A collaborative study with 19 coded compounds. Mutat. Res..

[48] Kamber M., Fluckinger-Isler S., Engelhardt G., Jaeckh R., Zeiger E. (2009). Comparison of the Ames II and traditional Ames test responses with respect to mutagenicity, strain specificities, need for metabolism and correlation with rodent carcinogenicity. Mutagenesis.

[49] de Umbuzeiro G.A., Rech C.M., Correia S., Bergamasco A.M., Cardenette G.H., Fluckiger-Isler S., Kamber M. (2010). Comparison of the Salmonella/Microsome Microsuspension Assay with the new Microplate Fluctuation Protocol for Testing the Mutagenicity of Environmental Samples. Environ. Mol. Mutagen..

[50] Fronza G., Campomenosi P., Iannone R., Abbondandolo A. (1992). The 4-nitroquinoline 1-oxide mutational spectrum in single stranded DNA is characterized by guanine to pyrimidine transversions. Nucleic Acids Res..

[51] U.S. Department of Health and Human Services Food and Drug Administration Center for Drug Evaluation and Research (CDER) (2015). Guidance for Industry: Waiver of In Vivo Bioavailability and Bioequivalence Studies for Immediate Release Solid Oral Dosage Forms Based on a Biopharmaceutics Classification System.

[52] (2008). CPMP/EWP/QWP/1401/98.

[53] Espuelas S., Plano D., Nguewa P., Font M., Palop J.A., Irache J.M., Sanmartín C. (2012). Innovative lead compounds and formulation strategies as newer kinetoplastid therapies. Curr. Med. Chem..

[54] Ruberte A.C., González-Gaitano G., Sharma A.K., Aydillo C., Encío I., Sanmartín C., Plano D. (2020). New Formulation of a Methylseleno-Aspirin Analog with Anticancer Activity towards Colon Cancer. Int. J. Mol. Sci..

[55] Berntssen M.H.G., Sundal T.K., Olsvik P.A., Amlund H., Rasinger J.D., Sele V., Hamre K., Hillestad M., Buttle L., Ørnsrud R. (2017). Sensitivity and toxic mode of action of dietary organic and inorganic selenium in Atlantic salmon (*Salmo salar*). Aquat. Toxicol..

[56] Nakatsuji M., Inoue H., Kohno M., Saito M., Tsuge S., Shimizu S., Ishida A., Ishibashi O., Inui T. (2015). Human Lipocalin-Type Prostaglandin D Synthase-Based Drug Delivery System for Poorly Water-Soluble Anti-Cancer Drug SN-38. PLoS ONE.

[57] Fujimura H., Komasaka T., Tomari T., Kitano Y., Takekawa K. (2016). Nanosuspension formulations of poorly water-soluble compounds for intravenous administration in exploratory toxicity studies: In vitro and in vivo evaluation. J. Appl. Toxicol..

[58] Petit C., Bujard A., Skalicka-Woźniak K., Cretton S., Houriet J., Christen P., Carrupt P.A., Wolfender J.L. (2016). Prediction of the Passive Intestinal Absorption of Medicinal Plant Extract Constituents with the Parallel Artificial Membrane Permeability Assay (PAMPA). Planta Med..

[59] Liu W., Okochi H., Benet L.Z., Zhai S.D. (2012). Sotalol Permeability in Cultured-Cell, Rat Intestine, and PAMPA System. Pharm. Res..

[60] Markovic B.D., Vladimirov S.M., Cudina O.A., Odovic J.V., Karljikovic-Rajic K.D. (2012). A PAMPA Assay as Fast Predictive Model of Passive Human Skin Permeability of New Synthesized Corticosteroid C-21 Esters. Molecules.

[61] Hallifax D., Turlizzi E., Zanelli U., Houston J.B. (2012). Clearance-dependent underprediction of in vivo intrinsic clearance from human hepatocytes: Comparison with permeabilities from artificial membrane (PAMPA) assay, in silico and caco-2 assay, for 65 drugs. Eur. J. Pharm. Sci..

[62] Serra H., Mendes T., Bronze M.R., Simplício A.L. (2008). Prediction of intestinal absorption and metabolism of pharmacologically active flavones and flavanones. Bioorg. Med. Chem..

[63] Takahashi K., Suzuki N., Ogra Y. (2017). Bioavailability Comparison of Nine Bioselenocompounds In Vitro and In Vivo. Int. J. Mol. Sci..

[64] Mukkavilli R., Yang C., Singh Tanwar R., Ghareeb A., Luthra L., Aneja R. (2017). Absorption, Metabolic Stability, and Pharmacokinetics of Ginger Phytochemicals. Molecules.

[65] Letelier M.E., Izquierdo P., Godoy L., Lepe A.M., Faúndez M. (2004). Liver Microsomal Biotransformation of Nitro-aryl Drugs: Mechanism for Potential Oxidative Stress Induction. J. Appl. Toxicol..

[66] Fasinu P., Bouic P.J., Rosenkranz B. (2012). Liver-based in vitro technologies for drug biotransformation studies—A review. Curr. Drug Metab..

[67] Yilmazer M., Stevens J.F., Deinzer M.L., Buhler D.R. (2001). In vitro biotransformation of xanthohumol, a flavonoid from hops (*Humulus lupulus*), by rat liver microsomes. Drug Metab. Dispos..

[68] Xiong H., Turner K.C., Ward E.S., Jansen P.L., Brouwer K.L. (2000). Altered hepatobiliary disposition of acetaminophen glucuronide in isolated perfused livers from multidrug resistance-associated protein 2-deficient TR(-) rats. J. Pharmacol. Exp. Ther..

[69] Asha S., Vidyavathi M. (2009). *Cunninghamella*-a microbial model for drug metabolism studies-a review. Biotechnol. Adv..

[70] Zagrodzki P., Paśko P., Domínguez-Álvarez E., Salardón-Jiménez N., Sevilla-Hernández C., Sanmartín C., Bierła K., Łobiński R., Szpunar J., Handzlik J. (2022). Synthesis of novel organic selenium compounds and speciation of their metabolites in biofortified kale sprouts. Microchem. J..

[71] Huang S., Zhang Y., Zhong J., Pan Y., Cai S., Xu J. (2018). Toxicological profile and safety pharmacology of a single dose of fibroblast activation protein-α-based doxorubicin prodrug: In-vitro and in-vivo evaluation. Anticancer. Drugs.

[72] MacLachlan T.K., Milton M.N., Turner O., Tukov F., Choi V.W., Penraat J., Delmotte M.H., Michaut L., Jaffee B.D., Bigelow C.E. (2017). Nonclinical Safety Evaluation of scAAV8-RLBP1 for Treatment of RLBP1 Retinitis Pigmentosa. Mol. Ther. Methods Clin. Dev..

[73] MacArthur Clark J. (2017). The 3Rs in research: A contemporary approach to replacement, reduction and refinement. Br. J. Nutr..

[74] Ragazzo P., Feretti D., Monarca S., Dominici L., Ceretti E., Viola G., Piccolo V., Chiucchini N., Villarini M. (2017). Evaluation of cytotoxicity, genotoxicity, and apoptosis of wastewater before and after disinfection with performic acid. Water Res..

[75] Ames B.N., Shigenaga M.K., Gold L.S. (1993). DNA lesions, inducible DNA repair, and cell division: Three key factors in mutagenesis and carcinogenesis. Environ. Health Perspect..

[76] Słoczyńska K., Powroźnik B., Pękala E., Waszkielewicz A.M. (2014). Antimutagenic compounds and their possible mechanisms of action. J. Appl. Genet..

[77] Ames B.N., McCann J., Yamasaki E. (1975). Methods for detecting carcinogens and mutagens with the Salmonella/mammalian-microsome mutagenicity test. Mutat. Res..

[78] Szemerédi N., Kincses A., Rehorova K., Hoang L., Salardón-Jiménez N., Sevilla-Hernández C., Viktorová J., Domínguez-Álvarez E., Spengler G. (2020). Ketone- and Cyano-Selenoesters to Overcome Efflux Pump, Quorum-Sensing, and Biofilm-Mediated Resistance. Antibiotic.

[79] Kandaş N.O., Randolph C., Bosland M.C. (2009). Differential effects of selenium on benign and malignant prostate epithelial cells: Stimulation of LNCaP cell growth by noncytotoxic, low selenite concentrations. Nutr. Cancer.

[80] Verma A., Atten M.J., Attar B.M., Holian O. (2004). Selenomethionine stimulates MAPK (ERK) phosphorylation, protein oxidation, and DNA synthesis in gastric cancer cells. Nutr. Cancer.

[81] Benassi J.C., Barbosa F.A.R., Candiotto G., Grinevicius V.M.A.S., Filho D.W., Braga A.L., Pedrosa R.C. (2021). Docking and molecular dynamics predicted B-DNA and dihydropyrimidinone selenoesters interactions elucidating antiproliferative effects on breast adenocarcinoma cells. J. Biomol. Struct. Dyn..

[82] Gajdács M., Handzlik J., Sanmartín C., Domínguez-Álvarez E., Spengler G. (2018). Rákellenes és efflux pumpa gátló hatású szelénvegyületek ADME tulajdonságainak becslése számítógépes módszerrel [Prediction of ADME properties for selenocompounds with anticancer and efflux pump inhibitory activity using preliminary computational methods]. Acta Pharm. Hung..

