# Supplementary material for: Pharmaceutical and Safety Profile Evaluation of Novel Selenocompounds with Noteworthy Anticancer Activity
Source: Pharmaceutics. 2022 Feb 6;14(2):367. doi: 10.3390/pharmaceutics14020367 (PMC8875489; doi:10.3390/pharmaceutics14020367)
Supplement: Supplementary file 1 [file pharmaceutics-14-00367-s001.zip › pharmaceutics-1568465-supplementary.pdf]

pharmaceutics

Supplementary Materials: Pharmaceutical and Safety Profile Evaluation of Novel Selenocompounds with Noteworthy Anticancer Activity

Małgorzata Anna Marć, Enrique Domínguez-Álvarez, Gniewomir Latacz, Agata Doroz-Płonka, Carmen Sanmartín, Gabriella Spengler and Jadwiga Handzlik

1. Material and Methods: detailed descriptions

1.1. List of reagent, bacteial strains and equipment

1.1.1. Experimental aqueous solubility tests - equipment and reagents

Methanol for UV spectrometry was acquired from Chempur (Piekary Śląskie, Poland); 0.2 mm silica-coated aluminum TLC plates from Merck (Darmstadt, Germany). Double distilled water was obtained at laboratory distillery. Quantitative hard filters "Macherey-Nagel MN 619 de" and glass bottles (6 mL and 12 mL) with polyethylene stopper were acquired from "Bolesławiec". Spectroscopic UV/Vis absorption spectrum of the samples were taken using a spectrometer Jasco V-530, at the wavelength range from 190 to 1100 nm. The light sources used in the V-530 were a deuterium (D2) lamp (190 to 350 nm) for the UV region and a halogen (WI) lamp (340 to 1100 nm) for the VIS/NIR region.

1.1.2. Reagents and materials for chemical stability test

Sodium hydroxide, hydrochloric acid and solvents for TLC analysis were acquired from Chempur (Piekary Śląskie, Poland). Anhydrous methanol and TLC plates were obtained from Merck (Darmstadt, Germany).

1.1.3. Reagents, materials and equipment for PAMPA test (membrane permeability evaluation)

Water for HPLC analysis was acquired from Merck (Darmstadt, Germany). Boric acid (0.4 M) and 0.1 M sodium borate decahydrate (both HPLC grade, in the water) were acquired from Beckman Coulter (USA). Potassium phosphate monobasic, sodium phosphate dibasic, sodium chloride and potassium chloride were acquired from Chempur (Piekary Śląskie, Poland). NaCl and HCl for CE were acquired from Fluka - Sigma Aldrich (Seelze, Germany). Caffeine and norfloxacin were acquired from Sigma-Aldrich (St. Louis, MO, USA). Precoated PAMPA Plate System Gentest™ was acquired from Corning (Bradford, MA, USA).

PAMPA test was performed using the capillary electrophoresis (CE) system P/ACE MDQ (Beckman Coulter, Fullerton, CA, USA), controlled by 32 Karat Software version 8.0. This CE system was equipped with a diode-array detector (DAD) and analytical uncoated fused-silica capillary with total length of 60 cm (50.2 cm to detection window) and internal diameter 75 μm), acquired from Beckman [38,41].

1.1.4. Reagents and equipment for the biotransformation with recombinant human liver microsomes

Recombinant, pooled and commercially available human liver microsomes (HLMs adult male & female) were acquired from Sigma-Aldrich (St. Louis, USA). TRIS-HCl buffer (0.1 M, pH 7.4) and NADPH Regeneration System were acquired from Promega (Madison, WI, USA). Pure DMSO and methanol for LC-MS/MS analysis were provided by Sigma-Aldrich (Seelze, Germany).

The biotransformation by human liver microsomes was evaluated by LC-MS/MS experiments. In the LC-MS/MS, the HPLC was performed using an Agilent 1100 HPLC system (AgilentTechnologies, Waldbronn, Germany) equipped with a XbridgeTM C18 analytical column (2.1 × 30 mm, 3.5 µM, Waters, Dublin, Ireland). The mobile phase was an elution gradient of acetonitrile and water, with a 0.1 % of formic acid. MS spectra were taken at an Applied Biosystems MDS Sciex API 200 triple quadrupole mass analyzer (Concord, Ontario, Canada) with an electro-spray ionization (ESI) interface between HPLC and MS. After HLMs biotransformation, probes were analyzed using an UPLC-MS/MS system conformed by a Waters Acquity UPLC (Waters, Milford, CT, USA) coupled to a Waters TQD mass spectrometer (electrospray ionization mode ESI-tandem quadrupole).

1.1.5. Reagents, bacterial strain and equipment for Ames microplate mutagenicity assay

Regarding reagents, ampicillin was acquired from Polfa Tarchomin S.A. (Warszawa, Poland); 4-nitroquinoline-*N*-oxide (NQNO), DMSO and bromocresol purple were acquired from Sigma-Aldrich (Seelze, Germany). Doxorubicin hydrochloride was acquired from Cayman chemical (Ann Arbor, Michigan 48108 USA). Beef extract, *L*-histidine monochloride, *D*-biotin were acquired from Bioshop (Burlington, Canada); whereas peptone from casein was acquired from Merck (Darmstadt, Germany). Ammonium sulfate, magnesium sulfate heptahydrate, potassium phosphate monobasic, potassium phosphate, trisodium citrate dehydrate, sodium chloride and *D*-glucose were acquired from Chempur (Piekary Śląskie, Poland).

Salmonella enterica serovar Typhimurium TA 100 strain was acquired from Xenometrix, Switzerland AG (Allschwil, Switzerland).

The inhibitory effect on the growth of *Salmonella* Typhimurium TA 100 strain of all tested selenocompounds was evaluated in the independent reference experiment on the basis of obtained OD_{600} values, which were measured on the microplate reader Perkin Elmer (EnSpire).

1.2. Determination of the aqueous solubility

1.2.1. Preparation of the saturated solutions of tested compounds

The saturated solutions (2 mL) of the tested Se-compounds in water were prepared at H_2O boiling point in small glass vials. Later, vials were covered to avoid evaporation and all solutions were left 1 day at 20° C, after when every solution was filtered. Finally, the concentration of the Se-compounds was determined through UV spectrophotometric measurements. To estimate correctly the concentration, calibration curves with known concentrations were prepared, and the saturated solutions were diluted till reaching a point within the range of the respective calibration curve.

1.2.2. Preparation of the calibration curve stock solutions for UV spectrometry

The stock solutions of 10 selenoesters (**EDA-26**, **EDA-71**, **EDA-73**, **EDA-74**, **EDA-93**, **EDA-109**, **EDA-117**, **EDA-119**, **EDA-120**, **EDA-122**) and the selenoanhydride **EDA-A6** were prepared in methanol (99% purity) at a 1 g/L concentration. Additionally, calibration solutions in the range from 0.0005 to 0.02 [g/L] were prepared for each Se-compound and their UV absorbance was determined to draw the corresponding calibration curve.

1.2.3. Sample preparation of saturated filtrates for UV spectrometry

The 100-fold diluted solutions of the saturated filtrates of the Se-compounds were prepared in the anhydrous methanol (99% purity), by addition of 70 µL of the corresponding methanol stock solution over anhydrous methanol till filling a 7 mL final volume. Afterwards, the absorption of abovementioned solutions was determined. Finally, the solutions were diluted with 99% methanol from 1 to 160 times, in function of the compound. The aim of this procedure was to determine the concentration of the Se-compounds

through the controlled dilution of the solution with unknown concentration till its UV absorbance fell within the range of the calibration solutions.

1.2.4. Solubility estimation

For each compound, the calibration curve was calculated through the UV absorbance measurements and the linear equation of the linear part of the curve was calculated. The absorbance of the dilution was then interpolated in this range, finding its concentration. To determine the solubility, this concentration was multiplied by the dilution factor, which was specific for each derivative. This action enabled the estimation of the *in vitro* solubility of the tested derivatives in saturated solutions. All the solubility values were the average value obtained from the 3 independent measurements.

1.3. PAMPA test: membrane permeability evaluation.

The passive transport and the membrane absorption of 5 selected selenocompounds (**EDA-58**, **EDA-71**, **EDA-119**, **EDA-122**, **EDA-A6**) were evaluated in the *in vitro* Parallel Artificial Membrane Permeability Assay (PAMPA). The protocol of the permeability assay was similar to the ones previously described [38,40,41].

1.3.1. Stock solutions and sample preparation for PAMPA test

Working solutions of the tested compounds at the concentration of the 50 µM in the phosphate saline buffer (PBS, pH 7.4) were prepared from the stocks in the pure dimethyl sulfoxide (DMSO) and stored at -20° C. Moreover, to draw the precise calibration curves for each tested (**EDA-58**, **EDA-71**, **EDA-119**, **EDA-122**, **EDA-A6**) and reference compounds (caffeine and norfloxacin), the set of the corresponding concentrations in PBS (2.5 µM, 5 µM, 7.5 µM, 10 µM, 20 µM, 50 µM, 100 µM, and 200 µM) were prepared.

1.3.2. Plate preparation

The Precoated PAMPA Plate System Gentest™ (Corning, Bradford, MA, USA) was used to perform permeability assay for the 5 selected Se-compounds. The essential part of this plate was its oil/lipid tri-layer artificial membrane, that does not contain excessive solvents [40]. PAMPA Plate System was brought to room temperature 30 min before use. The 96-well filter plate precoated with lipids was used as a permeation acceptor, whereas the 96-well receiver plate acted as the permeation donor plate. Therefore, the compound solutions were added to the wells (300 µL/well) of the receiver plate and PBS was added to wells (200 µL/well) of the precoated filter plate.

Then, the filter plate was coupled with the receiver plate and both were incubated at room temperature for five hours without agitation. After that time, the plates were separated carefully, 150 µL of the solutions from each well of both plates were transferred into the sterile eppendorf's, and then analyzed by capillary electrophoresis method (CE). Finally, the permeability coefficients (Pe, [cm/s]) of the tested compounds were estimated using the formula provided by the PAMPA Plate System manufacturer [38,41].

1.3.3. Capillary electrophoresis (CE) analytical method

The concentrations of the Se-compounds and of the references (norfloxacin, caffeine) were calculated in both the donor and acceptor compartments using the capillary electrophoresis (CE) system P/ACE MDQ (Beckman Coulter, Fullerton, CA, USA), controlled by 32 Karat Software version 8.0. This CE system was equipped with a diode-array detector (DAD) and analytical uncoated fused-silica capillary with total length of 60 cm (50.2 cm to detection window) and internal diameter 75 µm), acquired from Beckman [38,41].

1.3.4. Data analysis

Permeability, in terms of [cm/s] has been calculated according to **Scheme 1**. The obtained results were compared with the data achieved for a high permeable reference drug and a low permeable reference drug (caffeine and norfloxacin, respectively).

where:

$$Pe = \frac{-\ln \frac{1 - C_A(t)}{C_{eq}}}{A \cdot \left(\frac{1}{V_D} + \frac{1}{V_A} \right) \cdot t} \qquad C_{eq} = \frac{C_D(t) \cdot V_D + C_A(t) \cdot V_A}{V_D + V_A}$$

Scheme 1. Calculation of the *in vitro* permeability [cm/s]. A (filter area) cm^2 = 0.3; V_D (donor well volume) mL = 0.3; V_A (acceptor well volume) mL = 0.2; t (incubation time) seconds = 18000; $C_A(t)$ (compound concentration in acceptor well at time t); $C_D(t)$ (compound concentration in donor well at time t).

1.4. In vitro metabolic stability evaluation in microsomes

1.4.1. Microsomal metabolic stability protocol

For the microsomal stability assay, the selenoester **EDA-71** was selected. Before the experimental procedure, the HLMs and solutions necessary for preparing the regeneration fraction were stored at -80° C and TRIS buffer at 4° C. The $NADP^+$ regeneration fraction was prepared freshly, at the day of the experiment.

1.4.2. Preparation of the working solutions and microsomal regeneration fraction

In the first step of the experiment, after selection of the compounds, the 1000 μM working solution was prepared in the TRIS buffer (0.1 M, pH 7.5) from the 10 mM stocks in DMSO. Then, the fresh regeneration fraction was prepared from the 220 μL TRIS buffer, 25 μL of the "A" solution and 5 μL of the "B" solution.

1.4.3. Human liver microsomes and solvent controls

Further, two controls have been prepared: solvent control (142 μL of TRIS buffer with 8 μL of HLMs, 50 μL of the regeneration fraction and 200 μL of the pure methanol) and tested compound control (190 μL of TRIS buffer with 10 μL of the 1 mM tested compound solution and 200 μL of methanol). The biotransformation was carried out at the precise concentration of 1 mg/mL of HLMs.

1.4.4. Biotransformation performance

In the next step 132 μL of TRIS buffer were added into a sterile eppendorf. After that, 10 μL of 1000 μM solution of tested compound in TRIS buffer were added to the abovementioned eppendorf and then, 8 μL of HLMs. The final concentration of tested compound was 50 μM. After gently mixing on the vortex, reaction mixture was preincubated for 5 minutes at 37° C on the thermoblock. Then, in the further step, the reaction was initiated by adding 50 μL of the $NADP^+$ regeneration fraction and incubated at 37° C for 2 h. After that time, to terminate the reaction, the 200 μL of pure, cold methanol (for LC-MS/MS analysis) was added to probe, which contained the tested compound with HLMs. Moreover, the same amount of cold methanol was added into the control sample with HLMs. After that procedure, abovementioned probes were centrifuged at 14500 rpm for 10 minutes, which equals (in the centrifuge used) to 14129 RCF. Finally, 300 μL of supernatant was collected into a new sterile eppendorf and samples were analyzed by LC-MS/MS [44,45].

1.5. Safety profile evaluation - Ames microplate mutagenicity assay

Safety profile was evaluated by the means of *in vitro* microbiological methods – adapted to microplate Ames test, which applies *Salmonella enterica* serovar Typhimurium bacterial strains (the gold standard in mutagenicity research).

1.5.1. Compounds preparation

To evaluate the impact of tested compounds and of references on *Salmonella enterica* serovar Typhimurium (onwards *Salmonella* Typhimurium) TA-100 strain growth by means of the Ames test, the compounds or the reference were dissolved in the pure DMSO, to obtain the corresponding 10 mM stock solutions. Working solutions for tested compounds (25 μM and 250 μM concentrations) were prepared before the assay in DMSO.

1.5.2. Microplate Ames mutagenicity assay protocol

Prior to the experiment, *Salmonella* Typhimurium was cultured overnight (NB-2 liquid medium in the presence of 25 μg/mL ampicillin). At the day of the experiment, fresh dilutions of the tested compounds (25 μM and 250 μM), reference compound (methylseleninic acid) and standard mutagen (4-nitroquinoline-*N*-oxide at 2.5 μM and 12.5 μM) in DMSO were prepared. The positive control selected for the mutagenicity assays was NQNO, which causes point mutations at the genome. Specifically, it promotes G:C → A:T transitions in *Salmonella* Typhimurium TA-100 strain and in the other bacteria such as *Vibrio harveyi* and *Escherichia coli* [32,50].

The mutagenic potential of the tested derivatives was evaluated by the incubation of the abovementioned bacteria (deprived of the ability to synthesize the histidine) with specific concentrations of the tested compounds (for 90 min in the exposure medium), containing a limited amount of histidine (enough to provide approximately 2-3 cell divisions). The occurrence of reversion events to histidine prototrophy was observed through the bacterial growth in the indicator medium without histidine after 48 h of incubation in temperature of 37° C [45]. The final compound and the mutagenic agent concentrations in each single well of a 384 well plate were 1 μM and 10 μM for the compounds; and 0.1 μM and 0.5 μM for the mutagen. Lastly, both a manual count and a microplate reader were used to obtain the results. The medium control baseline (MCB) was estimated as the mean number of revertants in the medium control plus one standard deviation. Each fold increase bigger than two times the baseline level ≥ 2.0 was considered as a mutagen alert. All the experiment and data analysis were prepared according to the procedure described before by Flückiger-Isler *et al.* [47]. The assay was performed in triplicates to provide statistical robustness.

1.5.3. Mutagenicity data analysis

Herein, in the more sophisticated 384 - well plate microfluctuation Ames test, a special liquid medium (containing indicator dye, which changes colour from violet to yellow in the response to bacterial metabolism) has been used. The whole method can be easily automated. Moreover, the reading of the results is very simple (visual counting or/and microplate reader). The assay was performed in triplicates to provide statistical robustness [48,49]. The number of yellow wells (positive ones) wells out of 48 wells per replicate and dose was compared with the amount of spontaneous revertants obtained in the negative control with DMSO [48]. The average number of wells containing revertants per culture and concentration were calculated from the triplicate sections, and the increases above the zero dose were determined at each concentration of the tested compounds [47-48]. Triplicates were performed for each experiment, and results were provided in terms of mutagenic index (MI). This is defined as the quotient of the number of revertant colonies induced in a tested sample and the number of revertants in a negative control (media

with 1% DMSO). A compound was considered mutagenic when MI was above 2.0. Otherwise, if binomial B-values calculated according the manufacturer protocol was B ≤ 0.01, it indicated the occurrence of cytotoxic events [29].

1.5.4. Reference experiment performance - the impact of tested selenocompounds on the Salmonella Typhimurium growth

The concentrations of all the tested compounds were prepared as before in the Ames microplate assay. During the experiment, *Salmonella* Typhimurium TA100 strain was cultured overnight at the same conditions like in the Ames test. At the day of the experiment, in the every single well of the 96-well plate was placed 144 μL of NB-2 medium, containing *Salmonella* Typhimurium TA100 cell suspension with OD_{600} value = 0.1. Then, to each well (containing NB-2 medium) were added 6 μL of pre-prepared dilutions of the tested compounds (at concentrations of 25 μM and 250 μM), 6 μL of positive control (NQNO in 2 concentrations: 12.5 μM and 2.5 μM, methylseleninic acid and doxorubicin at concentrations of 25 μM and 250 μM) respectively. The final compound and mutagenic agent concentrations in each single well of a 96-well plate were 1 μM, 10 μM and 0.1 μM, 0.5 μM respectively. Also a negative control (150 μl of NB-2 medium alone), and the negative control with DMSO (144 μl of NB2 medium + 6 μl of pure DMSO) were prepared. Then, 96-well plates were incubated in the thermostatic chamber at 37° C for 20 h, on the shaker (250 rpm). Finally, the next day, the OD_{600} values were read for each sample.

References in Supplementary (same numeration than in manuscript)

29. Marć, M.A.; Domínguez-Álvarez, E.; Słoczyńska, K.; Żmudzki, P.; Chłoń-Rzepa, G.; Pękala, E. *In Vitro* Biotransformation, Safety, and Chemopreventive Action of Novel 8-Methoxy-Purine-2,6-Dione Derivatives. *Appl Biochem Biotechnol*, **2018**, *184*(1), 124-139.
32. Ames, B.N.; Lee, F.D.; Durston, W.E. An improved bacterial test system for the detection and classification of mutagens and carcinogens. *Proc Natl Acad Sci USA*, **1973**, *70*(3), 782-786.
38. Nasim, M.J.; Witek, K.; Kincses, A.; Abdin, A.Y.; Żesławska, E.; Marć, M.A.; Gajdács, M.; Spengler, G.; Nitek, W.; Latacz, G.; Karczewska, E.; Kieć-Kononowicz, K.; Handzlik, J.; Jacob, C. Pronounced activity of aromatic selenocyanates against multidrug resistant ESKAPE bacteria. *New J Chem*, **2019**, *43*(15), 6021-6031.
40. Bujard, A.; Voirol, H.; Carrupt, P.A.; Schappler, J. Modification of a PAMPA model to predict passive gastrointestinal absorption and plasma protein binding, *Eur J Pharm Sci*, **2015**, *77*, 273-278.
41. Latacz, G.; Lubelska, A.; Jastrzębska-Więsek, M.; Partyka, A.; Sobiło, A.; Olejarz, A.; Kucwaj-Brysz, K.; Satała, G.; Bojarski, A.J.; Wesołowska, A.; Kieć-Kononowicz, K.; Handzlik, J. In the search for a lead structure among series of potent and selective hydantoin 5-HT7 R agents: The drug-likeness in vitro study. *Chem Biol Drug Res*, **2017**, *90*(6), 1295-1306.
44. Kamiński, K.; Zagaja, M.; Łuszczki, J.J.; Rapacz, A.; Andres-Mach, M.; Latacz, G.; Kieć-Kononowicz, K. Design, Synthesis, and Anticonvulsant Activity of New Hybrid Compounds Derived from 2-(2,5-Dioxopyrrolidin-1-yl)propanamides and 2-(2,5-Dioxopyrrolidin-1-yl)butanamides, *J Med Chem*, **2015**, *58*(13), 5274–5286.
45. Sadek, B., Saad A.; Latacz, G.; Kuder, K.; Olejarz, A.; Karcz, T.; Stark, H.; Kieć-Kononowicz, K. Non-imidazole-based histamine H3 receptor antagonists with anticonvulsant activity in different seizure models in male adult rats. *Drug Des Devel Ther*, **2016**, *10*, 3879-3898.
47. Flückiger-Isler, S.; Baumeister, M.; Braun, K.; Gervais, V.; Hasler-Nguyen, H.; Reimann, R.; van Gompel, J.; Wunderlich, H.-G.; Engelhardt, G. Assessment of the performance of the Ames IITM assay: A collaborative study with 19 coded compounds. *Mutat Res*, **2004**, *558*(1-2), 181-197.
48. Kamber, M.; Fluckinger-Isler, S.; Engelhardt, G.; Jaeckh, R.; Zeiger, E. Comparison of the Ames II and traditional Ames test responses with respect to mutagenicity, strain specificities, need for metabolism and correlation with rodent carcinogenicity. *Mutagenesis*, **2009**, *24*(4), 359-66.
49. Umbuzeiro, G. de A.; Rech, C.M.; Correia, S.; Bergamasco, A.M.; Cardenette, G. H.; Fluckiger-Isler, S.; Kamber, M. Comparison of the Salmonella/Microsome Microsuspension Assay with the new Microplate Fluctuation Protocol for Testing the Mutagenicity of Environmental Samples. *Environ Mol Mutagen*, **2010**, *51*(1), 31-38.
50. Fronza, G.; Campomenosi, P.; Iannone, R.; Abbondandolo, A. The 4-nitroquinoline 1-oxide mutational spectrum in single stranded DNA is characterized by guanine to pyrimidine transversions. *Nucleic Acids Res*, **1992**, *20*(6), 1283-1287.
